# Metabolic Regulation of Macrophages in Health and Disease

**DOI:** 10.1002/mco2.70868

**Published:** 2026-07-22

**Authors:** Shan Huang, Yu Zhang, Jiali Min, Jiahui Yang, Yuchen Li, Hao Zhang, Shanshan Liu

**Affiliations:** ^1^ National Clinical Research Center for Metabolic Diseases Key Laboratory of Diabetes Immunology, and Department of Metabolism and Endocrinology Ministry of Education The Second Xiangya Hospital of Central South University Changsha Hunan China; ^2^ Furong Laboratory Changsha Hunan China

**Keywords:** immunometabolism, macrophages, macrophage polarization, metabolic diseases, metabolic regulation

## Abstract

Macrophages orchestrate immune responses through remarkable phenotypic plasticity, which is intrinsically linked to their ability to reprogram intracellular metabolic pathways in response to microenvironmental cues. While recent advances have highlighted the role of aberrant macrophage metabolism in diverse diseases, a systematic synthesis integrating both intracellular and extracellular metabolic signals remains lacking. This review provides a comprehensive framework for understanding how core metabolic pathways—glycolysis, the TCA cycle, oxidative phosphorylation (OXPHOS), fatty acid oxidation (FAO), and amino acid metabolism—are rewired during macrophage polarization under the orchestration of upstream signaling cascades, including NF‐κB, PI3K/AKT/mTOR, JAK–STAT, and MAPK. We examine how exogenous metabolites such as succinate, itaconate, lactate, and amino acids reciprocally regulate macrophage function and discuss tissue‐specific metabolic signatures of macrophage subsets—including alveolar macrophages （AMs), Kupffer cells (KCs), and tumor‐associated macrophages (TAMs)—in the context of obesity, Type 2 diabetes (T2D), metabolic dysfunction‐associated steatotic liver disease (MASLD), infections, autoimmune disorders, and cancer. We further evaluate emerging therapeutic strategies targeting macrophage metabolism, summarizing preclinical and clinical advances across signaling pathways, metabolic nodes, cytokines, and cell‐based therapies with detailed trial data. By integrating cell‐intrinsic metabolic circuitry with extracellular signals, this review establishes a theoretical foundation for metabolism‐targeted immunotherapies and identifies key knowledge gaps for future investigation.

## Graphical abstract

Extracellular metabolites drive metabolic rewiring of glucose, lipid, and amino acid pathways in macrophages, steering their polarization toward pro‐ or anti‐inflammatory phenotypes and shaping a wide range of disease processes. Macrophages from different tissues display distinct metabolic signatures, and therapeutic strategies that target these metabolic programs hold considerable promise for immunomodulation and precision clinical translation.

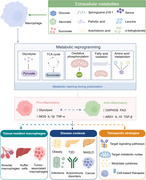

## 1 Introduction

Macrophages are indispensable sentinels of the innate immune system, residing in all mammalian tissues where they orchestrate host defense, maintain tissue integrity, and regulate metabolic homeostasis [1, 2]. Originating from either embryonic progenitors or bone marrow‐derived monocytes, these phagocytes exhibit extraordinary functional diversity—ranging from pathogen clearance and antigen presentation to wound healing and immune regulation [3]. For decades, in vitro studies have classically categorized macrophages into two extreme polarization states: proinflammatory (M1) macrophages induced by lipopolysaccharide (LPS) and interferon‐gamma (IFN‐γ) and anti‐inflammatory (M2) macrophages induced by interleukin‐4 (IL‐4) and IL‐13 [4]. This dichotomy has provided a foundational framework for understanding macrophage biology; however, it oversimplifies the complex phenotypic spectrum observed in vivo, where tissue‐resident macrophages (TRMs) such as AMs, KCs, and microglia display unique metabolic and functional signatures tailored to their specific microenvironments [5].

In recent years, the burgeoning field of immunometabolism has revolutionized our understanding of macrophage biology by revealing that cellular metabolism is not merely a housekeeping process but a critical determinant of immune cell fate and function [6 ^,^ 7]. Seminal studies have demonstrated that macrophage polarization is accompanied by profound metabolic reprogramming: proinflammatory macrophages shift from OXPHOS to aerobic glycolysis—a phenomenon reminiscent of the Warburg effect—to support rapid ATP production and biosynthetic demands, while anti‐inflammatory macrophages rely primarily on FAO and an intact tricarboxylic acid (TCA) cycle to sustain their long‐term anti‐inflammatory and tissue‐repair functions [8, 9, 10]. Key transcription factors such as hypoxia‐inducible factor 1 alpha (HIF‐1α), nuclear factor kappa B (NF‐κB), and peroxisome proliferator‐activated receptor gamma (PPARγ) orchestrate these metabolic switches by regulating the expression of glucose transporters, glycolytic enzymes, and lipid metabolism‐related genes [11, 12].

Despite remarkable progress in elucidating macrophage‐intrinsic metabolic pathways, several critical gaps remain. First, most mechanistic insights have been derived from reductionist in vitro systems using bone marrow‐derived macrophages (BMDMs) or cell lines, which fail to recapitulate the complex metabolic microenvironment in vivo—including nutrient fluctuations, oxygen tension, and intricate cellular crosstalk [13, 14]. Second, while the metabolic reprogramming of macrophages has been implicated in diverse pathologies, a systematic synthesis that integrates both cell‐intrinsic metabolic circuitry and cell‐extrinsic metabolic signals (such as dietary metabolites, microbiota‐derived products, and intercellular metabolic communication) is conspicuously lacking. Furthermore, the advent of single‐cell sequencing and spatial multiomics technologies has unveiled unprecedented heterogeneity among tissue‐resident and tumor‐TAMs, yet how this diversity arises from metabolic regulation remains incompletely understood [15, 16].

This review aims to synthesize current knowledge on macrophage metabolic regulation, with an emphasis on identifying common principles that recur across different physiological and pathological contexts. We begin by revisiting the metabolic foundations of classical macrophage polarization, delineating the distinct utilization of glucose, lipids, and amino acids in proinflammatory versus anti‐inflammatory states, and highlighting the key signaling pathways that govern these metabolic programs. Subsequently, we explore cell‐extrinsic metabolic regulation, examining how exogenous metabolites shape macrophage phenotypes through receptor‐mediated signaling and epigenetic modifications. A major focus is the tissue‐specific metabolic heterogeneity of macrophages, where we synthesize recent single‐cell and spatial transcriptomic findings to elucidate how unique microenvironmental cues program distinct metabolic signatures in AMs, KCs, TAMS, and other specialized subsets. Finally, rather than cataloging disease associations, we examine how recurring themes—metabolic flexibility, context‐dependent metabolite functions, and the integration of intracellular and extracellular signals—manifest across obesity, T2D, metabolic dysfunction‐aMASLD, autoimmune disorders, and cancer. By identifying these cross‐disease principles, we aim to provide a conceptual framework that informs therapeutic development and highlights key knowledge gaps.

## 2 Macrophage Diversity and Metabolic Foundations

### 2.1 Heterogeneity of TRMs

Macrophages represent one of the most plastic cell populations within the immune system, exhibiting remarkable functional diversity that is tightly coupled to their tissue of residence. Developmentally, macrophages originate from two principal lineages: embryo‐derived TRMs, which arise from yolk sac erythromyeloid progenitors and fetal liver monocytes, and bone marrow‐derived monocyte‐derived macrophages (MDMs), which are recruited to tissues during inflammation and injury. Embryo‐derived macrophages—including AMs in the lung, KCs in the liver, microglia in the brain, and Langerhans cells in the skin—primarily function to maintain tissue integrity and regulate homeostasis. In contrast, MDMs are recruited to sites of infection or injury, where they differentiate into mature macrophages that orchestrate inflammatory responses, pathogen clearance, and subsequent tissue repair [6, 17, 18].

Recent advances in single‐cell RNA sequencing have dramatically expanded our understanding of macrophage heterogeneity, revealing novel subpopulations with unique molecular phenotypes within specific disease microenvironments. For instance, inflammatory and metabolically activated macrophages (iMAMs), characterized by high expression of ATF4, PDIA3, ACSL4, and CCL2, are significantly enriched in the adipose tissue of obese individuals and exacerbate diet‐induced obesity through proinflammatory effects [7]. Similarly, TREM2^+^ NASH‐associated macrophages (NAMs), which highly express TREM2 and GPNMB, exhibit transcriptomic signatures enriched in phagocytosis and lysosomal degradation pathways and have been shown to promote tumorigenesis and drive NLRP3 inflammasome activation in steatohepatitis [19]. Other specialized subsets include lipid‐associated macrophages (LAMs), which are involved in lipid metabolism and inflammatory responses; vascular‐associated macrophages (VAMs), which regulate vascular homeostasis; and sympathetic neuron‐associated macrophages (SAMs), which modulate neural signaling [20, 21, 22]. Multiple macrophage subsets identified via single‐cell RNA sequencing in recent years, together with their phenotypic features and biological functions, are summarized in Table 1. These findings underscore the extraordinary diversity of macrophage populations and highlight the need to understand how distinct tissue microenvironments shape their functional identities.

**TABLE 1:** Characteristics of macrophage subtypes identified via scRNA‐seq.

Phenotype	Markers (human)	Markers (mouse)	Functions	References
Microglia‐like cells	P2RY1, SALL1, TMEM119, C3	/	Interacts with neural crest cells and modulates their differentiation toward the melanocyte lineage	[23]
Proangiogenic macrophages (PraM)	MRC1, CD83, CD45	/	Promotes angiogenesis	[23]
Lipid‐laden macrophages (LLMs)	GPNMB, FABP5, HMOX1, SPP1, ARG	CD45, CD11B, PLIN2, GPNMB, FABP5, HMOX1, SPP1, ARG1	Transfers myelin‐derived lipids to glioblastoma cells to fuel tumor progression	[24]
APOE+CD163+TAM	APOE, CD163, C1QA, IGF1, CCL3	/	Promotes tumor epithelial–mesenchymal transition	[16]
TREM2^+^NASH‐associated macrophages (NAMs)	TREM2, MS4A7	TREM2, MS4A7, PF4, CD9, MMP12, GPNMB	Drives NLRP3 inflammasome activation	[19]
S100a9^hi^ macrophages	/	CCR2, LY6C, S100A9	Amplifies inflammatory responses and promotes fibrosis after myocardial ischemia‒reperfusion	[15, 25]
TREM2/APOE/C1Q‐positive macrophages	C1Q, TREM2, APOE	TREM2, ARG1	Suppresses immunity and serves as a potential prognostic biomarker for clear cell renal carcinoma recurrence	[26]
Inflammatory and metabolically activated macrophages (iMAMs)	ATF4, PDIA3, ACSL4, CCL2	ATF4, PDIA3, ACSL4, CCL2	Promotes inflammation and obesity	[7]
Metabolically active macrophages (MMacs)	FN1, MARCO, FCGR3A, TIMD4, NR1H3, GPNMB, CYP27A1, TREM2	REM2, PNMB, MARCO, TIMD4, LYVE1, FOLR2, CD16, CD206	Inhibits inflammation and regulates lipid metabolism	[27]

### 2.2 The Classical M1/M2 Paradigm: A Metabolic Perspective

Despite the highly continuous spectrum of macrophage heterogeneity observed in vivo, in vitro studies have historically categorized macrophages into two classical polarization states based on specific stimulation protocols: proinflammatory (M1) macrophages induced by LPS and IFN‐γ, and anti‐inflammatory (M2) macrophages induced by IL‐4 and IL‐13 [4, 28]. While this dichotomy represents an oversimplification of in vivo complexity, it has provided an invaluable framework for understanding the fundamental relationship between macrophage function and cellular metabolism.

The M1 and M2 polarization states are accompanied by profoundly distinct metabolic programs. Proinflammatory macrophages undergo a metabolic switch toward aerobic glycolysis, disrupted TCA cycle, and increased pentose phosphate pathway (PPP) flux, enabling rapid ATP production, biosynthetic precursor generation, and antimicrobial effector molecule synthesis [29, 30, 31]. In contrast, anti‐inflammatory macrophages primarily rely on OXPHOS and FAO to meet their sustained energy demands, supporting prolonged anti‐inflammatory and tissue‐repair functions [32, 33]. These divergent metabolic programs are not merely passive consequences of polarization but actively dictate macrophage functional capacity by controlling energy availability, biosynthetic potential, and the production of immunomodulatory metabolites.

The molecular mechanisms underlying these metabolic programs—including the regulation of glycolysis, TCA cycle remodeling, fatty acid metabolism, and amino acid utilization—will be systematically examined in Section 3. Additionally, the upstream signaling pathways that orchestrate these metabolic changes, such as NF‐κB, PI3K/AKT/mTOR, JAK–STAT, and MAPK, are discussed in detail in Section 4.

### 2.3 Limitations of in Vitro Models and the Need for Integrated Analysis

While significant progress has been made in elucidating the metabolic pathways that govern macrophage polarization using reductionist in vitro systems, it is essential to acknowledge the inherent limitations of these approaches when translating findings to physiological and pathological conditions in vivo [14]. Most studies investigating macrophage metabolism rely on homogeneous cell models—such as murine BMDMs or human THP‐1 cells—cultured under static, supraphysiological nutrient conditions that poorly mimic the complex in vivo microenvironment [34, 35]. Standard cell culture oxygen tension (approximately 18% O_2_) differs markedly from the physiological hypoxic conditions prevalent in most tissues, including the liver, adipose tissue, and tumor microenvironment (TME) [13, 36]. Moreover, in vitro systems lack the dynamic nutrient fluctuations, complex cytokine networks, and intricate cell‒cell interactions—including paracrine signaling, extracellular vesicle exchange, and direct contact with neighboring cells—that continuously shape macrophage metabolism and function in vivo [37, 38].

These limitations are particularly relevant when considering the metabolic heterogeneity of TRMs. For example, AMs residing in the unique high‐oxygen, low‐glucose, surfactant‐rich lung microenvironment exhibit metabolic features—such as reliance on OXPHOS for lipid processing—that cannot be recapitulated in conventional BMDM cultures [39, 40]. Similarly, KCs in the liver maintain their physiological phenotype through lipid metabolic pathways regulated by the transcription factors LXRα, RXRα, and PPARδ, which are modulated by the hepatic microenvironment in ways that in vitro models cannot fully capture [41, 42]. Therefore, integrating findings from reductionist in vitro studies with emerging data from single‐cell multiomics, organoid models, and precision‐cut tissue slices is essential for developing a comprehensive understanding of macrophage metabolism in health and disease. This integrated approach will be a central theme throughout this review.

## 3 Core Metabolic Pathways in Macrophage Polarization

The functional polarization of macrophages is fundamentally governed by the dynamic rewiring of intracellular metabolic networks. Far from being passive bystanders, metabolic pathways actively dictate cellular fate by providing energy, generating biosynthetic precursors, and producing signaling molecules that modulate gene expression and protein function. In this section, we systematically dissect the core metabolic pathways—glycolysis, the TCA cycle, OXPHOS, fatty acid metabolism, and amino acid metabolism—that distinguish proinflammatory (M1‐like) and anti‐inflammatory (M2‐like) macrophages, highlighting the emerging role of immunometabolites such as itaconate in shaping immune responses.

### 3.1 Glycolysis and the PPP

A hallmark of proinflammatory macrophage activation is a metabolic switch from OXPHOS to aerobic glycolysis, a phenomenon first observed in murine peritoneal macrophages over five decades ago [43, 44]. This metabolic reprogramming, analogous to the Warburg effect in cancer cells, enables rapid ATP production and supplies glycolytic intermediates for biosynthetic pathways [29, 30]. Upon LPS or IFN‐γ stimulation, macrophages upregulate the glucose transporter GLUT1 via HIF‐1α, facilitating increased glucose uptake [45]. Concurrently, the expression and activity of key glycolytic enzymes are profoundly altered. The PFKFB3 isozyme, which generates fructose‐2,6‐bisphosphate—a potent allosteric activator of phosphofructokinase‐1—is markedly upregulated, driving glycolytic flux [46]. Similarly, the M2 isoform of pyruvate kinase (PKM2) undergoes a switch from highly active tetramers to less active dimers. This dimeric PKM2 not only sustains glycolytic intermediate accumulation but also translocates to the nucleus, where it functions as a coactivator for HIF‐1α and signal transducer and activator of transcription 3 (STAT3), directly enhancing the transcription of glycolytic genes and proinflammatory cytokines such as IL‐1β [12].

The PPP, a glycolytic branch, is also differentially regulated during macrophage polarization. In proinflammatory macrophages, the oxidative phase of the PPP is selectively enhanced, generating NADPH for reactive oxygen species (ROS) production by NADPH oxidase 2 (NOX2), a critical mechanism for pathogen killing [47]. Conversely, the nonoxidative phase, which relies on sedoheptulose kinase CARKL, is suppressed. This inhibition directs glucose‐derived carbon toward the oxidative phase, further amplifying NADPH and ROS generation [48]. In contrast, anti‐inflammatory macrophages exhibit elevated CARKL expression, favoring the nonoxidative phase to produce ribose‐5‐phosphate, a precursor for nucleotide and UDP‐N‐acetylglucosamine (UDP‐GlcNAc) synthesis, which is essential for the glycosylation of M2 markers such as the mannose receptor (CD206) [49, 50].

### 3.2 TCA Cycle Remodeling and Itaconate Biology

Beyond flux changes in glycolysis, proinflammatory activation induces profound remodeling of the TCA cycle, creating a signature two‐break model. The first break occurs at isocitrate dehydrogenase, leading to citrate accumulation. Accumulated citrate is exported from the mitochondria via the citrate carrier SLC25A1 and can be used for fatty acid synthesis (FAS), prostaglandin production, or conversion to itaconate [51]. The second break occurs at succinate dehydrogenase (SDH), impairing the oxidation of succinate to fumarate and causing succinate accumulation [52]. Elevated succinate stabilizes HIF‐1α by inhibiting prolyl hydroxylase domain (PHD) enzymes, thereby sustaining the glycolytic program and promoting IL‐1β expression [52]. Notably, the increased succinate‐to‐α‐ketoglutarate (α‐KG) ratio itself serves as a metabolic checkpoint that favors proinflammatory gene expression.

The diversion of citrate to itaconate represents one of the most striking examples of metabolic pathway repurposing in immunity. Itaconate is synthesized from cis‐aconitate by the enzyme aconitate decarboxylase 1 (ACOD1, also known as IRG1), which is highly induced in proinflammatory macrophages [53]. Originally identified as an antimicrobial metabolite, itaconate has emerged as a potent immunoregulatory molecule. It exerts anti‐inflammatory effects through multiple mechanisms: (i) directly alkylating and activating the transcription factor nuclear factor erythroid 2‐related factor 2 (NRF2), leading to the expression of antioxidant genes [54]; (ii) inhibiting the NLRP3 inflammasome; and (iii) suppressing the phosphorylation of Janus kinase 1 (JAK1) and STAT6, thereby limiting the alternative activation of macrophages [55]. The itaconate derivative 4‐octyl itaconate has been widely used to recapitulate these anti‐inflammatory effects, highlighting the therapeutic potential of targeting this pathway.

In anti‐inflammatory macrophages, the TCA cycle operates as an intact, oxidative circuit. This integrity is essential for meeting the sustained ATP demands of these cells via OXPHOS. FAO and glutamine metabolism serve as primary anaplerotic sources, replenishing TCA intermediates without disrupting cycle flux [56, 57].

### 3.3 OXPHOS and Electron Transport Chain Dynamics

OXPHOS, the process by which the electron transport chain (ETC) generates ATP coupled to oxygen consumption, is the predominant energy source for anti‐inflammatory macrophages and TRMs under homeostatic conditions [39]. In these cells, a functional ETC, particularly Complexes I and II, supports efficient ATP synthesis. However, in proinflammatory macrophages, the role of the ETC is fundamentally repurposed. Upon LPS stimulation, the assembly and activity of ETC complexes are remodeled. While Complex II (SDH) activity is maintained, Complex I and III activities are modulated to favor mitochondrial ROS (mtROS) production over ATP synthesis [40]. The oxidation of accumulated succinate by SDH drives reverse electron transport at Complex I, generating a burst of mtROS that serves as a signaling molecule to stabilize HIF‐1α and promote IL‐1β transcription [58]. Pharmacological inhibition of SDH with dimethyl malonate attenuates this proinflammatory signature, underscoring the centrality of ETC remodeling in inflammatory macrophage function [58]. This shift from ATP synthesis to ROS generation represents a fundamental principle of metabolic reprogramming: the same machinery is adapted to meet distinct functional demands—bactericidal activity in one context and tissue repair in another.

### 3.4 Fatty Acid Metabolism: Synthesis Versus Oxidation

Lipid metabolism is a key discriminant between macrophage polarization states. Proinflammatory macrophages upregulate FAS. Mitochondrial uncoupling protein 2 (UCP2) activates fatty acid synthase (FASN), which not only supports membrane remodeling during phagocytosis but also promotes the activation of the NLRP3 inflammasome, thereby enhancing IL‐1β and IL‐18 secretion [59, 60]. In contrast, anti‐inflammatory macrophages are characterized by enhanced FAO. This is orchestrated by the transcription factor PPARγ and its coactivator PGC‐1β, which are induced downstream of IL‐4/STAT6 signaling [61, 62]. PARγ promotes the expression of the fatty acid transporter CD36, facilitating the uptake of exogenous fatty acids, while PGC‐1β drives the expression of mitochondrial FAO enzymes, including carnitine palmitoyltransferase 1 (CPT1) [63]. CPT1 catalyzes the rate‐limiting step of FAO: the transport of long‐chain fatty acids into the mitochondria for β‐oxidation. The resulting acetyl‐CoA fuels the TCA cycle, and the reducing equivalents (NADH and FADH_2_) support OXPHOS, providing the sustained energy required for prolonged anti‐inflammatory functions such as tissue repair and helminth defense [64].

### 3.5 Amino Acid Metabolism: Arginine, Glutamine, and Beyond

Amino acid metabolism is intimately linked to macrophage effector functions. Arginine metabolism exemplifies this dichotomy: proinflammatory macrophages express inducible nitric oxide synthase (iNOS), which converts arginine to nitric oxide (NO) and citrulline [65]. NO is a key antimicrobial molecule and contributes to inflammatory signaling. Conversely, anti‐inflammatory macrophages upregulate arginase 1 (ARG1), which hydrolyzes arginine to ornithine and urea [66]. Ornithine is further metabolized by ornithine decarboxylase (ODC) to produce polyamines (e.g., putrescine, spermidine), which are essential for cell proliferation and tissue repair, and by ornithine aminotransferase to generate proline for collagen synthesis [67].

Glutamine is the most abundant plasma amino acid and plays multiple roles. In proinflammatory macrophages, glutamine is metabolized via glutaminase 1 (GLS1) to glutamate and then to α‐KG, which can replenish the TCA cycle (anaplerosis) and support succinate production [68]. This pathway is crucial for sustaining the inflammatory response. However, in anti‐inflammatory macrophages, α‐KG derived from glutamine has a distinct function: it serves as a cofactor for Jumonji domain‐containing (Jmjd3) demethylases, promoting the demethylation of histone H3 lysine 27 (H3K27) at the promoters of M2 marker genes (e.g., *Arg1, Mrc1*), thereby facilitating epigenetic reprogramming toward an anti‐inflammatory phenotype [69]. Furthermore, glutamine contributes to the hexosamine biosynthetic pathway, generating UDP‐GlcNAc for protein glycosylation, which is critical for the surface expression and function of M2 receptors [49, 70, 71].

Other amino acids also play specialized roles. Serine and glycine fuel one‐carbon metabolism, supporting the production of S‐adenosylmethionine (SAM), the universal methyl donor for histone and DNA methylation, which regulates inflammatory gene expression [72, 73]. Tryptophan catabolism via indoleamine 2,3‐dioxygenase (IDO) produces kynurenine, which can promote anti‐inflammatory phenotypes and T‐cell suppression, particularly in the TME [74, 75].

**FIGURE 1:**
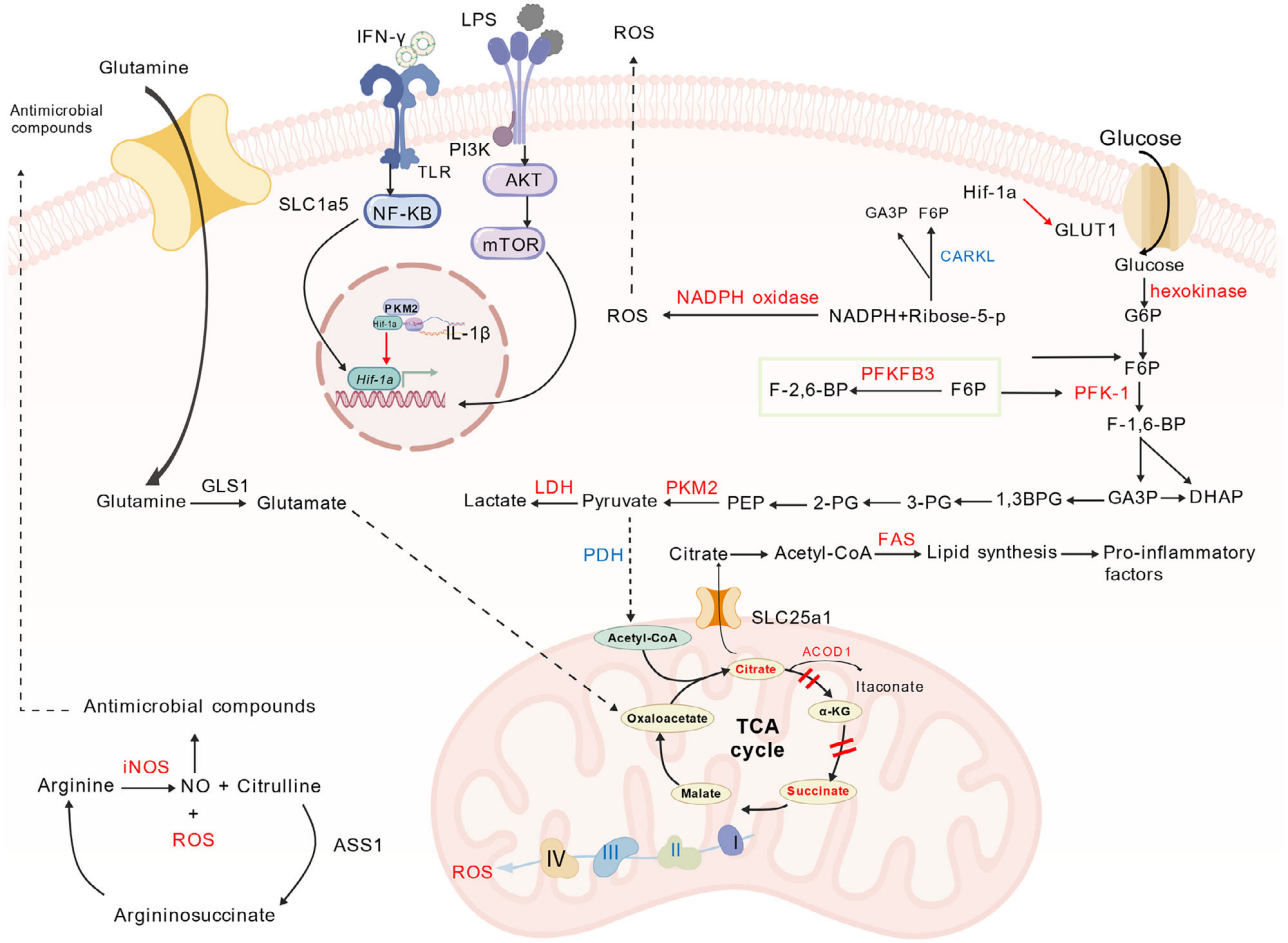
Cell‐intrinsic metabolic control of pro‐inflammatory macrophages. The pro‐inflammatory polarization induced by LPS/IFN‐γ initiates a shift toward aerobic glycolysis, driven by the upregulation of HIF‐1α, GLUT1, and glycolytic enzymes (PFKFB3 and PKM2). Disruption of the TCA cycle leads to the accumulation of succinate, which stabilizes HIF‐1α, and the diversion of citrate to itaconate. The oxidation of succinate through SDH produces mitochondrial ROS, which increase IL‐1β production, while the PPP provides NADPH for ROS protection. Furthermore, FAS promotes the production of pro‐inflammatory factors. Arginine metabolism transitions to iNOS‐mediated NO synthesis, and glutamine contributes to the production of α‐KG for succinate formation. These metabolic changes collectively increase inflammatory and antimicrobial responses. AKT, protein kinase; CoA, coenzyme A; DHAP, dihydroxyacetone phosphate; F‐1,6‐BP, fructose‐1,6‐bisphosphate; F‐2,6‐2P, fructose‐2,6‐bisphosphate; F6P, fructose‐6‐phosphate; G6P, glucose 6‐phosphate; GA3P, glyceraldehyde 3‐phosphate; GLUT1, glucose transporter 1; GS, glutamine synthetase; LDH, lactate dehydrogenase; PFK‐1, 6‐phosphofructokinase‐1; PFKFB3, 6‐phosphofructo‐2‐kinase/fructose‐2,6‐biphosphatase 3; PIK3, phosphoinositide 3‐kinase; SLC1A5, solute carrier family 1 member 5; 1,3BPG, 1,3‐bisphosphoglycerate; 2‐PG, 2‐phosphoglycerate.

**FIGURE 2:**
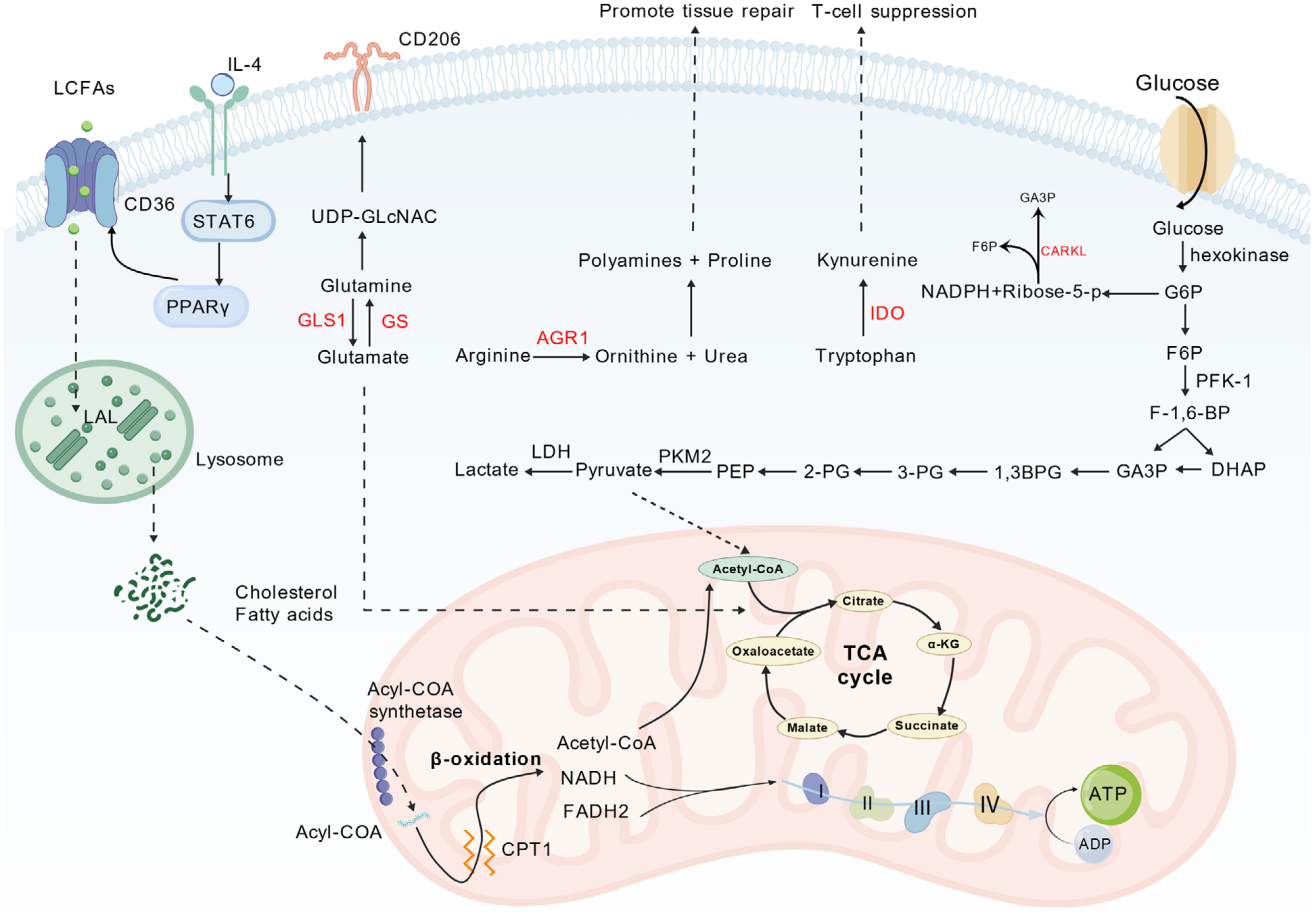
Cell‐intrinsic metabolic control of anti‐inflammatory macrophages. PPARγ enhances the uptake of fatty acids via CD36, whereas CPT1 facilitates mitochondrial β‐oxidation to produce acetyl‐CoA for the TCA cycle. This metabolic activity is further stimulated by the STAT6‐dependent IL‐4 signaling pathway. Anti‐inflammatory polarization also involves glutamine metabolism, where GLS1 catalyzes the formation of α‐KG, which enters the functional TCA cycle. GS produces UDP‐GlcNAc, which is crucial for the glycosylation of anti‐inflammatory markers such as CD206. ARG1 converts arginine into ornithine, aiding in tissue repair. Additionally, IDO‐mediated metabolism of tryptophan to kynurenine promotes macrophage polarization toward the anti‐inflammatory phenotype. These metabolic adaptations equip anti‐inflammatory macrophages with anti‐inflammatory and tissue‐repairing capabilities. ADP, adenosine diphosphate; ASS1, argininosuccinate synthetase; ATP, adenosinetriphosphate; CARKL, carbohydrate kinase‐like; CoA, coenzyme A; FADH2, flavin adenine dinucleotide; LAL, acid lipase; LCFAs, long‐chain fatty acids; LDH, lactate dehydrogenase; NADH, nicotinamide adenine dinucleotide; NADPH, nicotinamide adenine dinucleotide phosphate; PEP, phosphoenolpyruvate; SLC25a1, solute carrier family 25 member 1.

In summary, the metabolic reprogramming of macrophages is not a singular event but a coordinated rewiring of multiple, interconnected pathways. The choice between glycolysis and OXPHOS, the integrity of the TCA cycle, the balance between FAS and oxidation, and the specific utilization of amino acids collectively define the activation state and functional capacity of macrophages. This review systematically summarizes the characteristics of intracellular metabolic reprogramming during macrophage polarization toward pro‐ and anti‐inflammatory phenotypes, with a focus on alterations in key metabolic pathways and their critical regulators (Figure 1, 2 and Table 2). This intricate metabolic control provides a rich landscape for therapeutic intervention in diseases characterized by macrophage dysfunction.

**TABLE 2:** Core metabolic pathway remodeling in macrophage polarization.

Metabolic pathway	Key node	Proinflammatory (M1)	Anti‐inflammatory (M2)	Key regulators
Glucose metabolism	Glucose uptake	**↑↑↑**	→	GLUT1, HIF‐1α
	Glycolysis	↑↑↑ (Aerobic)	↓	PFKFB3, PKM2
	Pentose phosphate pathway	↑↑ (Oxidative)	↑↑ (Nonoxidative)	G6PD, CARKL [45, 46, 48]
TCA cycle	Cycle integrity	Broken	Intact	IDH, SDH
	Citrate	↑↑↑	→	SLC25a1
	Succinate	↑↑↑	→	SDH [52, 76, 77]
Oxidative phosphorylation	OXPHOS activity	↓ (to ROS)	↑↑	ETC complexes
	Mitochondrial ROS	↑↑↑	→	SDH, Complex I [40, 58]
Lipid metabolism	Fatty acid synthesis	↑↑	↓	FASN, UCP2
	Fatty acid oxidation	↓	↑↑	PPARγ, CPT1, CD36 [32, 60, 62]
Amino acid metabolism	Arginine	iNOS ↑ (NO)	ARG1 ↑ (ornithine)	iNOS, ARG1
	Glutamine	Catabolism ↑	Synthesis/catabolism ↑	GLS1, GS
	α‐Ketoglutarate	Supports succinate	Epigenetic	Jmjd3 [78, 79, 80]
Energy output	ATP production efficiency	Low (rapid)	High (sustained)	Glycolysis vs. OXPHOS [40]

*Note*: ↑↑↑, marked increase; ↑↑, moderate increase; ↑, increase; ↓, decrease; →, no significant change compared with resting/unpolarized macrophages.

Several themes emerge from this analysis of core metabolic pathways. First, metabolic flexibility—the ability of macrophages to switch between energy sources depending on availability and demand—underpins their functional plasticity. Second, metabolic intermediates play dual roles: succinate stabilizes HIF‐1α to promote inflammation, citrate fuels itaconate synthesis, and itaconate itself feeds back to limit inflammatory responses. Third, the same pathway can have different functional consequences depending on context: glycolysis supports rapid ATP production in proinflammatory macrophages but is dispensable for anti‐inflammatory polarization if OXPHOS remains intact. These principles, rather than any single pathway, form the conceptual foundation for understanding how metabolism shapes macrophage function.

## 4 Signaling Pathways Orchestrating Macrophage Metabolic Reprogramming

Macrophage metabolic reprogramming and phenotypic polarization depend on the integration of complex signaling networks that sense and respond to microenvironmental cues. Core pathways—including NF‐κB, JAK–STAT, PI3K/AKT/mTOR, and MAPK—are selectively activated during macrophage activation, triggering downstream kinase cascades that converge on key transcription factors. These molecular switches enable macrophages to translate extracellular signals into specific metabolic programs and immune functions. Understanding how these pathways orchestrate metabolic remodeling is essential for deciphering macrophage biology in health and disease.

### 4.1 NF‐κB Signaling Pathway

NF‐κB serves as a master regulator of innate immune responses, driving proinflammatory macrophage polarization and amplifying inflammatory cascades following pathogen recognition [11]. The NF‐κB family comprises five members: RelA (p65), RelB, c‐Rel, p50, and p52, which form various homo‐ or heterodimers. Notably, only RelA, RelB, and c‐Rel possess transcriptional activation domains, meaning that dimers containing these subunits can directly initiate target gene transcription [81]. Under steady‐state conditions, NF‐κB remains sequestered in the cytoplasm through interaction with inhibitory IκB proteins. The IκB kinase (IKK) complex—comprising IKKα, IKKβ, and the regulatory subunit IKKγ—controls this inhibitory interaction, with IKKβ recognized as the rate‐limiting kinase in the canonical NF‐κB pathway [82].

Upon recognition of pathogen‐associated molecular patterns such as LPS by Toll‐like receptors (TLRs) on the macrophage surface, receptor conformational changes recruit the adaptor protein MyD88, sequentially activating downstream IRAK, TRAF6, and TAK1 kinases [83]. This cascade phosphorylates the IKK complex, triggering its activation. Activated IKKβ then specifically phosphorylates IκB proteins at conserved N‐terminal sites, marking them for ubiquitination and proteasomal degradation. Liberated NF‐κB translocates to the nucleus, where it binds target promoters to initiate the transcription of inflammatory genes and key regulators such as HIF‐1α, thereby promoting proinflammatory metabolic reprogramming [84, 85].

### 4.2 AKT/mTOR Signaling Pathway

The PI3K–AKT–mTOR pathway integrates metabolic and inflammatory signals to fine‐tune macrophage responses [86]. Unlike the proinflammatory role of NF‐κB, the activation status of this pathway determines macrophage polarization direction in a context‐dependent manner: pathway activation generally promotes anti‐inflammatory phenotypes, while inhibition skews cells toward proinflammatory states [87].

Upstream signals—including those from TLR4, cytokine receptors, chemokine receptors, and Fc receptors—activate PI3K, which catalyzes phosphorylation of membrane phospholipid PIP2 to generate the second messenger PIP3. PIP3 recruits AKT and PDK1 to the membrane, facilitating AKT conformational change and exposing its phosphorylation sites. Concurrently, mTOR complexes are recruited, cooperating with PDK1 to achieve full AKT activation [88]. Activated AKT phosphorylates and inhibits the TSC complex, a negative regulator of mTORC, thereby initiating mTORC signaling. Active mTORC then phosphorylates downstream effectors to remodel cellular metabolic networks, establishing specific polarization phenotypes.

The AKT‐mTOR pathway regulates macrophage energy metabolism by modulating glucose metabolism and glycolytic enzyme activity, providing the metabolic foundation for polarization [89]. As a key nutrient sensor, mTORC couples intracellular nutritional status with polarization signals, enabling metabolic adaptation across diverse microenvironments. For example, in gastric cancer, SIGLEC11 promotes TAM polarization by regulating glucose metabolic reprogramming via the AKT–mTOR pathway, enhancing the immunosuppressive microenvironment and accelerating tumor progression [90]. Conversely, in atherosclerosis, a high‐protein diet inhibits macrophage mitophagy by activating mTOR signaling, exacerbating inflammation and plaque formation [91].

Beyond metabolism, this pathway regulates phagocytosis, autophagy, and inflammatory cytokine secretion. Its dysregulation is implicated in infectious diseases, metabolic disorders, tumors, and cardiovascular diseases [92], making it an attractive therapeutic target.

### 4.3 JAK–STAT Signaling Pathway

The JAK–STAT pathway mediates signals from numerous cytokines, regulating immune responses, inflammation, and cancer. When cytokines bind transmembrane receptors, receptor conformational changes recruit JAK kinases, triggering receptor phosphorylation. Phosphorylated receptors recruit specific STAT proteins, promoting their phosphorylation and dissociation. Activated STAT proteins dimerize and translocate to the nucleus, initiating target gene transcription [93]. In mammals, seven STAT family members exhibit differential activation patterns that underlie macrophage functional plasticity [94].

STAT1, STAT2, and STAT5 synergistically drive proinflammatory polarization. STAT1 not only induces proinflammatory cytokine expression but also reshapes cellular metabolism by binding glycolytic gene promoters, establishing a positive feedback loop between metabolism and inflammation that exacerbates conditions such as acute lung injury [95]. Citrulline can block JAK2–STAT1 signaling to inhibit proinflammatory macrophage activation [96]. STAT2 promotes mitochondrial proliferation and dysfunction through Drp1 phosphorylation, shifting mitochondria from ATP synthesis toward ROS production and activating NF‐κB to amplify inflammatory cytokine transcription [97]. IFN‐α activates the JAK2–STAT2 axis, forming a complex with IRF9 that directly binds the Gsdmd promoter, upregulating GSDMD expression and inducing pyroptosis [98]. STAT5 regulates oxidative stress and epigenetics: mitochondrial superoxide activates STAT5, which inhibits the TCA cycle and enhances chromatin accessibility at STAT5 target genes. In atherosclerosis, STAT5 inhibition blocks macrophage differentiation into Trem2^+^Gpnmb^+^ foam cells [99].

STAT3 and STAT6 promote anti‐inflammatory and TAM polarization. In the TME, CCL2‐secreting fibroblasts induce TAM polarization by activating JAK–STAT3 signaling, promoting immune evasion and tumor progression [100]. Conversely, itaconate and its derivatives block JAK1 and STAT6 phosphorylation, inhibiting TAM polarization and offering potential therapeutic targets [55]. Notably, JAK–STAT3 signaling can also promote proinflammatory polarization in certain contexts, such as 8:2 difluoroalkylphosphate diester‐induced hepatotoxicity [101], highlighting the pathway's context‐dependent functions.

### 4.4 MAPK Signaling Pathway

The MAPK family—including p38, JNK, and ERK—serves as a crucial signaling hub in cellular responses to exogenous stimuli. Through a conserved phosphorylation cascade, activated MAPKs phosphorylate cytoplasmic substrates and translocate to the nucleus to modify transcription factors, reshaping gene expression profiles [102]. Each subfamily plays distinct roles in macrophage polarization.

p38 MAPK mediates inflammatory responses and drives proinflammatory polarization through multiple mechanisms. Exosomal miR‐155‐5p from macrophages infected with hypervirulent *Klebsiella pneumoniae* activates p38 signaling by targeting MSK1, promoting proinflammatory polarization and exacerbating lung injury [103]. p38 also amplifies inflammation by activating inflammasome pathways: a high‐salt environment activates SGK1 via Slc6a12, upregulating p38 signaling and triggering NLRP3 inflammasome assembly through NF‐κB p65 nuclear translocation [104].

The MAPK/ERK pathway plays central roles in tumor development. In the TME, tumor cell‐conditioned media induces Fbxo38 expression in macrophages, activating ERK1/2 and IRF4 signaling to promote TAM‐specific gene expression and enhance immunosuppressive phenotypes [105]. Beyond cancer, this pathway regulates lipid metabolism disorders and atherosclerosis. Porphyromonas gingivalis induces oxidative stress via TLR4, activating MAPK/ERK signaling and mediating FOXO3 phosphorylation and degradation, upregulating scavenger receptor 1 expression, and promoting lipid uptake and programmed necrosis in macrophages [106].

JNK signaling typically promotes proinflammatory polarization. Its excessive activation skews macrophages toward proinflammatory phenotypes, while JNK inhibition alleviates inflammation and facilitates tissue repair. Polarized titanium‐based piezoelectric composites suppress macrophage inflammatory responses by downregulating MAPK/JNK signaling, inducing anti‐inflammatory polarization. In infectious bone defect repair, Icam1^+^ macrophages regulate bone regeneration; an iron‐doped barium titanate nanocarrier system targeting these cells reshapes energy metabolism by activating JAK2–STAT3 and inhibiting MAPK/JNK pathways, promoting a prorepair phenotype [107]. JNK may also synergize with p38 to drive proinflammatory polarization: in cerebral hemorrhage, melanocortin 4 receptor activation inhibits both JNK and p38 phosphorylation via an AMPK‐dependent pathway, reducing proinflammatory polarization and alleviating neuroinflammation [108]. In the TME, mannose‐modified M1 mitochondria increase ROS levels to activate NF‐κB p65, p38, and JNK pathways, reprogramming TAMs toward proinflammatory states and enhancing antitumor immunity [109].

In summary, the NF‐κB, PI3K/AKT/mTOR, JAK–STAT, and MAPK pathways form an integrated signaling network that translates microenvironmental cues into macrophage metabolic reprogramming. Extensive crosstalk among these pathways enables precise tuning of immune responses: NF‐κB and MAPK converge on inflammatory gene expression, PI3K/AKT/mTOR couples nutrient sensing with oxidative metabolism, and JAK–STAT regulates amino acid utilization and mitochondrial function. Through these mechanisms, signaling pathways directly shape the metabolic landscapes that define macrophage functional phenotypes. Understanding this signaling–metabolism interface has important therapeutic implications, with pathway‐targeted inhibitors already showing promise in preclinical and clinical studies. In the following section, we explore how cell‐intrinsic metabolic circuits—the downstream effectors of these pathways—execute and sustain macrophage phenotypic specification.

The four signaling pathways discussed above illustrate another unifying principle: signal integration and pathway crosstalk. NF‐κB and MAPK converge on inflammatory gene expression; PI3K/AKT/mTOR couples nutrient sensing with oxidative metabolism; JAK–STAT regulates amino acid utilization and mitochondrial function. This signaling network enables macrophages to integrate diverse environmental cues—pathogens, cytokines, nutrients, and metabolites—into coherent metabolic and functional responses. The context‐dependent outcomes of pathway activation—for example, STAT3 promoting immunosuppression in tumors but inflammation in other settings—underscore the importance of understanding signaling in its full microenvironmental context.

## 5 Tissue‐Specific Metabolic Signatures of Macrophages

Macrophage polarization and effector functions depend on dynamic reprogramming of intracellular metabolic networks. Remodeling glycolysis, fatty acid metabolism, and amino acid metabolism is now recognized as a key driver of phenotypic conversion [110]. Resting macrophages primarily use the TCA cycle and OXPHOS to maintain homeostasis. During proinflammatory polarization, metabolism shifts from OXPHOS to aerobic glycolysis to meet increased biosynthetic demands, with the PPP supplying ribose and NADPH for anabolic reactions [45]. In contrast, FAO serves as the primary energy source for anti‐inflammatory macrophages [111]. However, most insights into macrophage metabolism come from reductionist in vitro systems, and it is important to acknowledge their limitations when translating findings to in vivo conditions. This is especially critical for interpreting the metabolic characteristics of TRMs.

First, studies on macrophage metabolism rely heavily on in vitro culture systems [14]. While convenient, these models do not fully capture the complexity of the in vivo microenvironment. Oxygen tension in standard culture differs from the physiological hypoxic conditions in tissues such as liver, adipose tissue, and tumors [13, 36]. Nutrient concentrations in culture media are static and supraphysiological, lacking the dynamic fluctuations of glucose, fatty acids, and amino acids in vivo [34]. In vitro systems also cannot replicate the complex cytokine networks and cell‒cell interactions present in living organisms. In vivo, macrophages communicate with neighboring cells—hepatocytes, adipocytes, endothelial cells, and other immune cells—through paracrine signaling, extracellular vesicles, and direct contact, all of which shape metabolic reprogramming [37, 38]. Thus, conclusions from in vitro studies may not fully reflect the metabolic state and functional responses of macrophages in their native tissue environment.

Second, in vitro studies predominantly use homogeneous cell models such as mouse BMDMs or human THP‐1 cells [35]. Although valuable for studying basic mechanisms, these models lack the heterogeneity of in vivo macrophages. Advances in single‐cell RNA sequencing and spatial multiomics have identified numerous TRM subpopulations with unique molecular phenotypes and functions. These include VAMs that regulate vascular homeostasis, SAMs that modulate neural signaling, and LAMs involved in lipid metabolism and inflammation [20, 21, 22]. These subsets exhibit metabolic profiles tailored to their tissue‐specific functions. For example, LAMs rely on enhanced FAO to adapt to lipid‐rich environments, a feature that cannot be recapitulated in conventional BMDM or THP‐1 cultures.

Given the inherent heterogeneity of macrophages in vivo, traditional in vitro models capture only a fraction of macrophage phenotypes and cannot fully simulate the metabolic adaptability of tissue‐resident populations. Understanding these limitations is essential for interpreting research findings and bridging the gap between experimental systems and physiological conditions. With this foundation, the following discussion focuses on tissue‐specific macrophages, highlighting how the tissue microenvironment and functional requirements shape their distinctive metabolic phenotypes.

### 5.1 Lung Macrophage Subtypes

AMs, the core innate immune cells of the lung, originate from hematopoietic progenitors during embryonic development. Postnatally, they maintain homeostasis through local self‐renewal, during inflammation or injury, they can be replenished by bone marrow‐derived monocytes [112]. AMs reside in alveolar spaces and septa, forming the lung's primary immune barrier. They clear inhaled particles, surfactant components, and apoptotic cells. Upon infection, injury, or tumor development, AMs detect microenvironmental signals and regulate immune responses. Their dysfunction is linked to pulmonary infections, COPD, and lung cancer [113].

The lung microenvironment—high oxygen, low glucose, and abundant surfactant‐derived lipids—shapes the distinctive metabolic phenotype of AMs. Under homeostasis, AMs rely on OXPHOS and FAO, with OXPHOS crucial for lipid processing. When OXPHOS is impaired, lipid metabolism declines, triggering cholesterol accumulation and cellular stress, leading to cell cycle arrest and reduced cell numbers [40]. Glycolytic activity in steady‐state AMs remains low, with mitochondrial respiration driven by FAO. This metabolic pattern is regulated by PPAR‐γ, which promotes lipid uptake and maintains mitochondrial function, and is controlled by microenvironmental signals including epithelial‐derived GM‐CSF [114]. Notably, unlike the traditional view that proinflammatory macrophages rely on glycolysis, AMs during pulmonary infection still depend on OXPHOS to drive inflammatory cytokine production. This reflects an adaptive strategy evolved in response to the lung microenvironment [39].

Interstitial macrophages (IMs) reside in the pulmonary parenchyma, primarily originating from monocytes. They are distributed near blood vessels and airways, regulating local immune responses and maintaining tissue homeostasis [21]. IM development and polarization are regulated by signals from vascular endothelial cells. TGF‐β1 from pulmonary endothelial cells directs monocyte maturation into IMs by activating the TGF‐β receptor axis. Deficiencies in this pathway impair IM development and reduce cell numbers, suggesting that endothelial‐derived TGF‐β is critical for IM maintenance [115]. Endothelial cells also shape IM function through a paracrine–metabolic–epigenetic axis. Rspondin3 from endothelial cells promotes glutamine catabolism and increases α‐KG in IMs via Wnt/β‐catenin signaling. As a cofactor for TET2, this metabolite drives DNA hydroxymethylation, polarizing IMs toward an anti‐inflammatory phenotype and promoting resolution of pulmonary inflammation [116].

### 5.2 Liver Macrophage Subtypes

The liver contains two distinct macrophage populations: KCs of embryonic origin and MDMs. KCs, identified by high Tim4 and Clec4F expression, play key roles in pathogen clearance, tissue repair, and hepatic homeostasis. Under physiological conditions, KC self‐renewal is independent of bone marrow‐derived cells. During inflammation, resident KCs die and are replaced by infiltrating MDMs, which secrete inflammatory mediators and contribute to lipid accumulation and fibrosis, driving liver disease progression [117].

KCs are characterized by potent phagocytic capacity, enabled by scavenger receptors that recognize polyamines and phosphatidylserine on senescent cells and debris [118]. This phagocytic function relies on energy from OXPHOS and FAO, providing ATP for phagosome formation, cytoskeletal rearrangement, and lysosomal acidification.

Lipid metabolic homeostasis is also central to the KC phenotype. KCs overexpress lipid metabolism genes, including CD36, CPT1A, CPT2, and ACSL1, regulated by LXRα, RXRα, PPARδ, and PPARγ [41]. Under homeostasis, KCs take up lipids via CD36, drive cholesterol efflux and FAO through LXR/PPARδ signaling, and clear lipids via lysosomal lipolysis, maintaining an anti‐inflammatory phenotype. Rbpj deficiency in macrophages promotes lipid uptake and FAO in Ly6Clo monocytes by upregulating CD36 and suppressing proinflammatory activation [42].

In contrast to KCs, infiltrating MDMs exhibit hyperglycolysis, suppressed OXPHOS, and succinate accumulation, stabilizing HIFs. These cells downregulate lipid oxidation genes and prioritize rapid energy production via glycolysis while releasing proinflammatory cytokines including TNF‐α, IL‐1β, and IL‐6, driving hepatic inflammation [119]. HIF‐2α exacerbates lysosomal stress in KCs under MASH conditions, promoting inhibitory phosphorylation of TFEB via mTOR and ERK, downregulating lysosomal and phagocytic genes, and impairing phagocytic function [120]. Under pathological stress such as NASH, KC phagocytic function and lipid homeostasis are disrupted, leading to chemokine secretion that recruits MDMs. Notably, HIF‐2α has subtype‐specific roles: in MDMs, it does not regulate lysosomal function but amplifies inflammation by upregulating ANT2, inducing mitochondrial permeability transition, and promoting ROS production and proinflammatory activation [120].

### 5.3 TAM Subtypes

TAMs are the most abundant immune cells in the TME and exhibit high functional plasticity. Regulated by microenvironmental signals, TAMs switch between proinflammatory and anti‐inflammatory states, with corresponding antitumor or protumor activities [121]. Single‐cell and spatial multiomics have revealed their complex origins, spatial distribution, and dynamic polarization during tumor progression.

TAMs derive from TRMs and MDMs. As tumors progress, TRMs decrease and migrate to the periphery, while MDMs are recruited to the tumor core, regulated by chemokines and the TME. In early tumor formation, TRMs cluster around tumor cells, promoting immune evasion by inducing epithelial–mesenchymal transition and recruiting regulatory T cells [122].

TAMs exhibit temporal heterogeneity correlated with tumor stage. In early development, TAMs polarize toward a proinflammatory phenotype with enhanced aerobic glycolysis, mitochondrial fragmentation (shifting from ATP to ROS production), and overexpression of TNF‐α, IL‐1β, iNOS, CXCL10, and CCL2. This state enables immune surveillance and clearance of nascent tumor cells [123].

As tumors progress, factors including hypoxia, tumor‐derived cytokines (IL‐4, IL‐10, CSF‐1), and exosomes induce metabolic reprogramming in TAMs. Lipid metabolism is enhanced, with FAO becoming the primary energy source [32]. Amino acid metabolism, particularly glutamine, becomes highly active to support biosynthesis and anti‐inflammatory mediator production. UDP‐GlcNAc flux increases, providing substrates for protein O‐glycosylation. Upregulation of OXCT1 induces succinate accumulation, elevating H3K4me3 at the Arg1 promoter and driving its transcription toward a protumor phenotype [124]. Hedgehog signaling promotes STAT6 O‐glycosylation, enhancing its transcriptional activity and driving immunosuppressive TAM polarization [125].

This phenotypic switch highlights TAM plasticity: they can be reprogrammed toward protumor or antitumor states depending on microenvironmental signals.

### 5.4 Other Macrophage Subtypes

Cardiac macrophages are the most abundant immune cells in cardiac tissue. Based on CCR2 expression, they are classified as CCR2^−^ (yolk sac‐derived) and CCR2^+^ (monocyte‐derived). During embryonic development, CCR2^−^ macrophages promote endothelial migration toward vascular perfusion zones by secreting IGF, mediating coronary artery dilation [126]. At this stage, the hypoxic uterine environment leads macrophages to rely on lactate metabolism via OXPHOS. After birth, cardiac macrophage metabolism shifts toward FAO [127].

The spleen contains multiple specialized macrophage populations. Red pulp macrophages express Spi‐C and clear senescent red blood cells, maintaining iron homeostasis [128]. Marginal zone macrophages express CD204 and MARCO, regulating B cell localization. Marginal metallophilic macrophages participate in antiviral and antibacterial defense. Tingible body macrophages in germinal centers clear apoptotic lymphocytes; defects in this function are linked to autoimmune diseases [129].

Renal macrophages (RMs) expand and mature in parallel with kidney development. Lineage tracing shows that they originate from embryo‐derived RMs (EMRMs) rather than the yolk sac. This lineage self‐renews for at least 1 year; postnatally, bone marrow‐derived RMs (BMRMs) continuously replenish the population, with both lineages contributing equally under steady‐state conditions [130]. EMRMs exhibit a CX3CR1^high^ F4/80^high^ phenotype, while BMRMs are CCR2^high^CD11c^high^. The CX3CR1/CX3CL1 axis is essential for RM development and self‐renewal; CX3CR1 knockout reduces RM numbers from birth. EMRMs show enhanced glycolytic and mitochondrial respiratory capacity and superior immune complex clearance compared with BMRMs [131].

While intracellular metabolism is crucial for macrophage function, these processes do not occur in isolation. Macrophages are constantly influenced by external signals—nutrient availability, oxygen tension, cell‒cell interactions, and secreted metabolites. These extracellular signals act as upstream regulators, dynamically modulating intracellular metabolism and shaping functional responses. In the following sections, we focus on how extracellular metabolic signals regulate macrophage metabolism and function.

## 6 Cell‐Extrinsic Metabolic Signals Shaping Macrophage Responses

**FIGURE 3:**
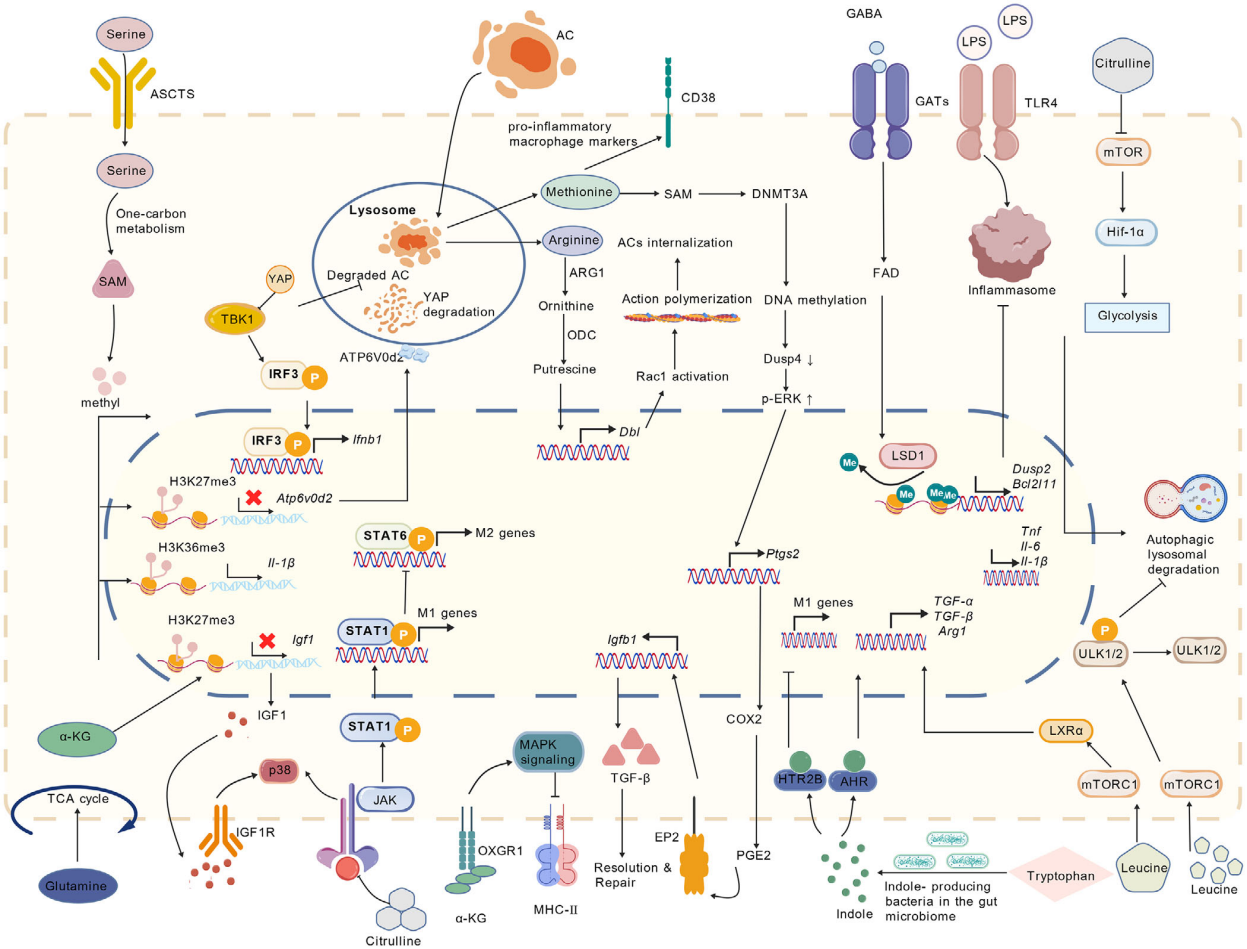
Metabolic regulation of the macrophage response by amino acids and their derivatives. A reduction in exogenous serine leads to proinflammatory polarization through decreased levels of SAM (via the IGF1–p38 pathway), while SAM‐dependent H3K36me3 facilitates the production of IL‐1β in response to LPS. Additionally, a lack of serine enhances antiviral immunity by inhibiting H3K27me3, which activates the ATP6V0d2–YAP–TBK1 signaling pathway. Arginine promotes anti‐inflammatory macrophage‐mediated efferocytosis through its conversion into ornithine and putrescine, whereas methionine can activate the Ptgs2/PGE2–TGFβ pathway, promoting inflammation resolution and tissue repair. GABA inhibits NLRP3 inflammasome activation by influencing the histone demethylation of Bcl2l11 and Dusp2 through the succinate–FAD–LSD1 signaling pathway. Tryptophan metabolites, such as indole, induce immunosuppressive macrophage phenotypes via AhR signaling, affecting conditions such as colitis and cancer. Leucine activates mTORC1, leading to anti‐inflammatory macrophage polarization, but excessive intake can hinder autophagy and increase inflammation. Citrulline has anti‐inflammatory properties through two mechanisms: it inhibits the mTOR–HIF1α–glycolysis pathway to reduce aging‐related inflammation and directly binds to JAK2 to suppress JAK2–STAT1 signaling, thereby downregulating proinflammatory genes. α‐KG plays a dual role in regulating macrophages by promoting anti‐inflammatory polarization through H3K27 demethylation at the promoters of anti‐inflammatory marker genes while inhibiting M1 activation through the suppression of IKKβ activation. ASCTs, alanine–serine–cysteine transporters; ATP6V0d2, ATPase H^+^ transporting V0 subunit d2; *Bcl2l11*, B‐cell lymphoma 2 like 11; COX2, cyclooxygenase 2; *Dbl*, diffuse B‐cell lymphoma; DNMT3A, DNA methyltransferase 3A; DUSP4, dual specificity phosphatase 4; EP2, prostaglandin E receptor 2; ERK, extracellular signal‐regulated kinase; FAD, flavin adenine dinucleotide; GATs, GABA transporters; HTR2B, 5‐hydroxytryptamine receptor 2B; *Igfb1*, insulin‐like growth factor binding protein 1; IRF3, interferon regulatory factor 3; LSD1, lysine specific demethylase 1; LXRα, liver X receptor α; MHC‐II, major histocompatibility complex Class II; ODC, ornithine decarboxylase; OXGR1, oxoglutarate receptor 1; P, phosphorylation; PGE2, prostaglandins E2; *Ptgs2*, prostaglandin–endoperoxide synthase 2; RAC1, ras‐related C3 botulinum toxin substrate 1; TBK1, TANK binding kinase 1; *Tnf*, tumor necrosis factor; ULK1/2, unc‐51 like kinase 1/2; YAP, yes‐associated protein.

Macrophages, pivotal components of the immune system, exhibit metabolic adaptations and functions that are considerably influenced by extrinsic environmental factors, including diet, microbiota, and metabolites [132]. A comprehensive understanding of how macrophages respond to external environmental factors is imperative for the development of novel therapeutic strategies and interventions. For example, by modulating metabolic processes, treating diseases such as obesity, diabetes, cardiovascular disease and cancer may be possible. Importantly, however, this field of research is still in its infancy. Below, we summarize the roles and molecular mechanisms of core extracellular metabolites in macrophage polarization (Figure 3 and Table 3).

**TABLE 3:** Core extracellular metabolites mediating macrophage activation and function.

Metabolites	Changes in cell‐extrinsic environment	Function	References
Glucose	Increased	Promotes M1 inflammation Inhibits M2 polarization and anti‐inflammation	[133, 134]
Fructose	Increased	Promotes M1 inflammation but inhibited M1 polarization in TME	[135, 136]
Citrate	Increased	Reprograms TAMs to polarize into M1	[137, 138]
Itaconate	Increased	Inhibits M1 proinflammatory cytokine production	[55]
Succinate	Increased	Varies among different tissues	[57, 139, 140]
D‐lactate	Increased	Reprograms M2 to M1	[141]
Sphingosine d18:1	Increased	Promotes inflammatory factors secretion	[142]
Sphingosine‐1‐phosphate	Increased	Promotes M1 inflammation	[143]
Sphingolipid	Increased	Promotes differentiation to decidual macrophages and enhances phagocytosis	[144]
Palmitic acid	Increased	Activates NF‐κB signaling	[145]
Arachidonic acid	Increased	Inhibits macrophage phagocytosis Reprograms decidual macrophages to polarize into M1	[146, 147]
Serine	Decreased	Contributes to M1 polarization Inhibits M2 polarization	[72]
Arginine	Increased	Promotes M2‐mediated continuous efferocytosis and injury resolution	[67]
Methionine	Decreased	Suppresses M1 polarization	[148]
Gamma‐aminobutyric acid	Increased	Decreases IL‐1β Promotes M2 polarization and IL‐10 expression	[149, 150]
Tryptophan	Increased	Reprograms M1 to TAMs	[74, 151]
Leucine	Increased	Contributes to M2 polarization Inhibits macrophage autophagy	[152, 153]
Citrulline	Decreased	Decreases macrophage proinflammatory gene expression	[154]
α‐ketoglutarate	Increased	Reprograms M1 to M2	[155, 156]

### 6.1 Carbohydrates and Their Metabolites

Carbohydrates, which are important energy sources and structural components in organisms, play a significant role in the immune system, especially in regulating the function of macrophages. Glucose, fructose, and intermediates of carbohydrate metabolism are the focal points of research in this field at present.

#### 6.1.1 Glucose

Glucose is the primary source of energy for cells, which is obtained from food and enters the body to be oxidized and broken down to produce ATP and support a wide range of physiological activities. It also plays an important role in regulating macrophage responses.

The proinflammatory response of proinflammatory macrophages is increased in the high‐glucose state, but their phagocytic and bactericidal functions are impaired. The underlying mechanism of impaired phagocytosis and bactericidal function in proinflammatory macrophages remains to be elucidated. Impaired phagocytosis has been shown to lead to the accumulation of ROS in the mitochondria, which, in turn, activates the NLRP3/caspase‐1 pathway to cleave GSDMD proteins [157]. GSDMD proteins play pivotal roles in cellular pyroptosis. After cleavage, the N‐terminus of the GSDMD protein can form transmembrane pores, leading to the release of inflammatory cytokines such as IL‐1β and a robust inflammatory response, ultimately leading to proinflammatory polarization of RAW264.7 macrophages and inflammatory cell death—namely, pyroptosis [158]. A recent study revealed that the expression of GLUT1 mRNA significantly increased in BMDMs in response to elevated glucose levels. This protein increases glucose uptake and glycolysis, subsequently activating mTOR signaling via GTP‐binding proteins. This process, in turn, results in increased production of TNF‐α and IL‐1β, thereby promoting an inflammatory response in proinflammatory macrophages [133]. However, compared with those from nondiabetic mice, peritoneal macrophages isolated from diabetic mice presented reduced expression of the glucose transporter protein GLUT1 and decreased glucose uptake and glycolysis [159]. Therefore, the mechanism underlying the increase in the inflammatory response in proinflammatory macrophages under high‐glucose conditions requires further investigation.

A decrease in the number of anti‐inflammatory macrophages and a concomitant reduction in their anti‐inflammatory function have been observed in high‐glucose environments. In normal tissue injury, the process of wound healing involves the infiltration of blood monocytes into the wound, where they differentiate into proinflammatory macrophages for the removal of foreign bodies and subsequently into anti‐inflammatory macrophages for tissue repair. However, another study revealed that in Type 2 diabetic mice, proinflammatory macrophages persist and remain proinflammatory while the transition to anti‐inflammatory macrophages is impaired, and anti‐inflammatory macrophages at the wound site not only decrease in number but also secrete anti‐inflammatory factors, such as IL‐10 and growth factors, resulting in delayed wound healing and an increased risk of infection [160].

#### 6.1.2 Fructose

Fructose is a natural monosaccharide widely found in fruits, honey, berries, and certain vegetables. As a significant source of carbohydrates in the diet, fructose can be absorbed and metabolized by the body, participating in various physiological processes. In recent years, increased intake of added fructose and refined sugars resulting from changes in dietary patterns has become a major factor affecting metabolic homeostasis.

Studies have revealed context‐dependent effects of fructose on macrophage polarization. In human monocytes and mouse BMDMs, fructose enhances the inflammatory response of proinflammatory macrophages to LPS stimulation. Mechanistically, fructose activates mTOR complex 1 (mTORC1) via the dihydroxyacetone phosphate receptor, leading to increased phosphorylation of its downstream target S6 ribosomal protein and promoting translational synthesis of inflammatory cytokines [135]. In contrast, within the TME, fructose acts as a signaling molecule that inhibits calcium ion release by enhancing the interaction between hexokinase 2 and the endoplasmic reticulum calcium channel ITPR3. This reduces intracellular calcium levels, ultimately suppressing proinflammatory macrophage polarization and promoting colorectal cancer growth [136]. Calcium signaling plays a pivotal role in proinflammatory macrophage polarization by activating pathways such as MAPK and STAT1 and regulating NLRP3 inflammasome activation.

These findings illustrate a key principle in immunometabolism: the same metabolite can exert opposing effects depending on the pathological context. In systemic inflammation, fructose amplifies proinflammatory responses, while in the TME, it suppresses them to support cancer progression. This context‐dependency highlights the need for careful consideration of metabolic interventions, as targeting fructose metabolism may have different outcomes in distinct disease settings. Further research is needed to elucidate the specific mechanisms by which fructose regulates macrophage function under various physiological and pathological conditions.

#### 6.1.3 Glycolytic Metabolites

Citrate is an intermediate product of the TCA cycle. Disruption of the TCA cycle in activated proinflammatory macrophages results in the accumulation of citrate in the cytoplasm, whereas in anti‐inflammatory macrophages, the cytoplasm is substantially depleted of citrate because of an intact TCA cycle and increased OXPHOS. The addition of exogenous citrate modulates macrophage polarization.

In one study, murine mononuclear macrophages were cultured with different concentrations of citrate, and citrate inhibited the polarization of both M1‐type macrophages and anti‐inflammatory‐type macrophages. Compared with anti‐inflammatory macrophages, proinflammatory macrophages with lower concentrations of citrate effectively inhibited their polarization, which was attributed to the more significant inhibitory effect of citrate on ATP synthesis and glycolytic enzyme activity in proinflammatory macrophages [161]. Conversely, another team incorporated citrate into a coculture system of pancreatic cancer cells and macrophages, confirming that citrate could inhibit the NF‐κB pathway, induce intracellular Ca2+ accumulation in pancreatic cancer cells, and consequently promote the expression of secreted proteins that are acidic and rich in cysteine. Following secretion, this protein acts on TLR4 on the membrane of macrophages through the transcription factor IRF3/7 to induce the expression of IFN‐stimulated genes [137]. This process, in turn, results in a shift in the anti‐inflammatory phenotype of TAMs to the proinflammatory phenotype, thereby inhibiting pancreatic cancer progression [138].

Citrate can be converted to itaconate by ACOD1. In proinflammatory macrophages, itaconate activates NRF2, which enhances the antioxidant response and drives the expression of activating transcription factor 3 (ATF3) while inhibiting the NLRP3 inflammasome to attenuate the inflammatory response in proinflammatory macrophages. In contrast, in anti‐inflammatory macrophages, itaconate and its derivative, 4‐octyl itaconic acid, impede anti‐inflammatory polarization by hindering the phosphorylation of JAK1 and STAT6, which are downstream of IL‐4 [55].

Succinate is also an intermediate of the TCA cycle. It accumulates in proinflammatory macrophages because of disruption of the TCA cycle and can trigger an inflammatory phenotype by stabilizing HIF1α. Research has shown that succinate accumulates in the lungs following intestinal ischemia–reperfusion, which is associated with an imbalance between succinate‐producing and succinate‐consuming bacteria in the intestinal tract. Furthermore, succinate can act on succinate receptor 1 (SUCNR1) on the membrane of AMs to activate the PI3K/AKT/HIF1α pathway, inducing the proinflammatory polarization of macrophages and exacerbating acute lung injury [57]. SSUCNR1 is expressed not only on AM membranes but also on macrophage membranes in intestinal and adipose tissue. A recent study revealed that the upregulation and activation of SUCNR1 in intestinal macrophages increased the expression of proinflammatory cytokines and proinflammatory marker genes in intestinal and peritoneal macrophages, whereas the lack of SUCNR1 shifted macrophages toward the M2 phenotype, which protected mice from colitis [140]. However, another study demonstrated that SUCNR1 activation promotes the polarization of adipose tissue macrophages toward the anti‐inflammatory phenotype and enhances the response to Type II cytokines (including IL‐4) via the PKA–CREB–KLF4 pathway [139]. Therefore, differences in tissue origin can result in different responses of macrophages to SUCNR1 activation.

Lactate is the end product of glycolysis. In mammals, lactate consists of two optical isomers: d‐lactate and l‐lactate. While l‐lactate is the main form of lactate present in the human body, d‐lactate is produced mainly during metabolism by certain microorganisms, which can lead to an accumulation of d‐lactate when certain intestinal bacteria are overgrown, such as Bifidobacterium spp. These two isomers are mirror images of the chemical structure but have very different functions in macrophages [162]. An endogenous “lactate clock” exists in proinflammatory macrophages that induce the expression of specific genes for a fixed period of time to maintain homeostasis. Proinflammatory polarization occurs when macrophages are exposed to stimuli such as LPS and IFN‐γ, leading to the reprogramming of OXPHOS to aerobic glycolysis and resulting in the accumulation of lactate, which can stimulate histone lysine lactylation 16–24 h later, acting as an epigenetic modification to directly stimulate gene transcription in chromatin to drive the expression of anti‐inflammatory‐like genes, such as the upregulation of ARG1 to produce ornithine for wound healing. Thus, the “lactate clock” contributes to the repair of collateral damage and infection that occurs in the host during proinflammatory polarization [163]. In contrast, the gut microbial small‐molecule metabolite d‐lactate converts TAMs from the anti‐inflammatory phenotype to the proinflammatory phenotype. d‐Lactate interacts with TLR2 and TLR9 on macrophage membranes, inducing the inhibition of the PI3K/Akt pathway and activation of the NF‐κB pathway to downregulate anti‐inflammatory associated genes (such as Arg1, Fizz, and IL10) while upregulating proinflammatory associated genes (such as TNF‐α, NOS and IL‐12). In addition, d‐lactate can enter the liver via the portal vein to increase the ability of KCs to remove pathogens from the body. Therefore, d‐lactate plays a key role in immunomodulation and tumor suppression in the treatment of hepatocellular carcinoma [141]. However, poor pharmacokinetics, low tumor accumulation, and targeting must be considered when exogenous d‐lactate is used in the clinic.

### 6.2 Lipids

Lipids are organic molecules characterized by their hydrophobic nature and can be categorized into a variety of types, such as sphingolipids, fatty acids, and glycerolipids. They play a variety of important roles in organisms, including energy storage, cell membrane construction, signal transduction, and metabolic regulation. Emerging evidence has shown that lipids are involved in the regulation of immune responses and inflammatory processes by influencing the phenotype and function of macrophages.

#### 6.2.1 Sphingolipids

Sphingolipids mainly include ceramide, sphingosine‐1‐phosphate (S1P), and sphingomyelins. LPS increases sphingolipid levels in M1‐like macrophages by inducing serine palmitoyltransferase long chain base subunit 2 (Sptlc2) expression. Sphingosine, the core structural backbone of sphingolipids, is required for the initiation of TLR4 signal transduction and the innate immune response through interactions with the TLR4 adaptor proteins MyD88 and TIRAP and the recruitment of MyD88 to the cell membrane of macrophages. The absence of sphinganine, which is caused by a myeloid cell‐specific deficiency in Sptlc2, disrupts TLR4‐driven inflammation in murine models of sepsis and melanoma [164]. Metabolomic analysis revealed that the level of sphingosine d18:1 is significantly increased in MASH patients. Sphingosine d18:1 specifically blocks the transcriptional activity of HIF2α in macrophages by inhibiting HIF2α binding to the ARNT subunit, thus promoting macrophage activation and ultimately aggravating MASH [142].

S1P is a bioactive sphingolipid that signals through five G protein‐coupled receptors (S1PR1–5), with S1PR1 and S1PR2 being the predominant isoforms expressed on macrophages. In ulcerative colitis, intestinal epithelial cells secrete S1P into the local microenvironment. S1P binding to S1PR1 on macrophage surfaces triggers dissociation of the heterotrimeric G protein into Gαi and Gβγ subunits. The Gαi subunit inhibits adenylyl cyclase, reducing cAMP levels and relieving PKA‐mediated inhibition of downstream proinflammatory cascades. Concurrently, the Gβγ subunit activates phospholipase C, generating inositol trisphosphate (IP3) and diacylglycerol (DAG). IP3 induces calcium release from endoplasmic reticulum stores, while DAG activates protein kinase C. These signals converge to activate the MAPK pathway (ERK1/2 and p38) and the NF‐κB pathway through IKK complex phosphorylation. This signaling cascade promotes proinflammatory macrophage polarization, induces the secretion of serum amyloid A1/3, and drives Th17 cell differentiation, exacerbating intestinal inflammation [143]. In contrast, S1P signaling through S1PR2 on macrophages has been shown to activate Rho kinase (ROCK) and inhibit macrophage migration, suggesting receptor‐subtype specificity in S1P‐mediated effects.

Glycosphingolipids (GSLs) are complex lipids that link sphingosine to fatty acid chains and oligosaccharides. GSLs are embedded in the outer leaflet of the plasma membrane, where they cluster with cholesterol and signaling proteins to form lipid rafts—dynamic microdomains that serve as platforms for signal transduction. GSLs regulate pattern recognition receptor signaling by modulating the lateral mobility and oligomerization of receptors within these rafts. Specifically, GSLs facilitate the clustering of TLR4 and CD14 upon LPS stimulation, enhancing receptor multimerization and recruitment of the downstream adaptors MyD88 and TRIF. GSLs also promote the assembly of C‐type lectin receptors such as Dectin‐1 into signaling complexes. Within the intracellular leaflet, GSL‐associated lipid rafts concentrate Src family kinases (Lyn, Hck, Fgr) and PI3K, facilitating their activation upon receptor engagement. This leads to phosphorylation of Akt and subsequent activation of NF‐κB, promoting macrophage phenotypic switching and inflammatory responses [144]. Additionally, GSLs have been shown to regulate phagocytosis by organizing F‐actin remodeling at the phagocytic cup through Rac1 and Cdc42 signaling.

These mechanistic insights reveal that sphingolipids are not merely structural components but also active signaling molecules that modulate macrophage function through specific receptor interactions, downstream kinase cascades, and membrane microdomain organization.

#### 6.2.2 Fatty Acyls

PA is the most abundant saturated fatty acid in the circulatory system. Elevated levels of PA are observed in the plasma of obese or diabetic individuals. This elevation is correlated with heightened atherosclerotic plaque vulnerability in the context of T2DM. Specifically, PA skews macrophages toward the proinflammatory proinflammatory phenotype by activating the TLR4 receptor, amplifying inflammation and driving the upregulation of delta‐like ligand 4 expression in macrophages, which in turn triggers senescence in vascular smooth muscle cells [165]. In addition, PA treatment stimulates macrophages to release exosomes with significantly elevated levels of miR‐3064‐5p. Exosomes secreted by PA‐challenged macrophages trigger an inflammatory response by targeting IκBα and activating NF‐κB signaling in recipient cells, including macrophages [145].

Arachidonic acid (AA) is a polyunsaturated fatty acid that is an important component of cell membrane phospholipids and a precursor of several inflammatory mediators (such as prostaglandins, leukotrienes, and thromboxanes). AA has been reported to regulate macrophage functions in ovarian cancer. Direct treatment of MDMs with AA triggers an increase in the intracellular Ca2+ level and subsequently triggers the activation of the ASK1–p38δ/α signaling axis, significantly increasing the transcription levels of proinflammatory factors, such as TNF‐α and IL‐6, and thereby amplifying the inflammatory response of macrophages [146]. A recent report suggested that spontaneous abortion is associated with aberrant lipid metabolism and macrophage‐mediated inflammatory responses. Excessive AA from decidual stromal cells is taken up by decidual macrophages via CD36 and the subsequent activation of the intracellular COX/PGE_2_ pathway, which in turn contributes to the conversion of decidual macrophages to a proinflammatory phenotype, ultimately leading to spontaneous abortion [147]. Taken together, these findings suggest that fatty acids play crucial roles in macrophage‐mediated inflammatory responses and metabolic homeostasis.

### 6.3 Amino Acids and Their Derivatives

Amino acids represent a class of vital nutrients that are indispensable for the promotion of optimal cell growth, differentiation, and function. They function as the fundamental constituents of protein synthesis and act as signaling molecules to regulate signaling pathways in vivo. They play important roles in the function of macrophages and in immune responses [80].

#### 6.3.1 Serine

Serine metabolism plays a crucial role in macrophage polarization and in modulating the immune response. The restriction of exogenous serine has been shown to promote macrophage polarization toward the proinflammatory phenotype and inhibit the STAT6‐mediated anti‐inflammatory phenotype. Mechanistic studies revealed that exogenous serine restriction leads to decrease levels of serine and its derived metabolite SAM, an important intracellular methyl donor, in macrophages. The downregulation of serine results in increased insulin‐like growth factor 1 (IGF1) expression by decreasing the level of histone methylation in the IGF1 promoter region. IGF1 activates the p38‐dependent JAK–STAT1 axis to promote proinflammatory polarization and inhibit STAT6‐mediated anti‐inflammatory activation [72]. However, another study suggested that serine restriction results in a reduced capacity of proinflammatory macrophages to produce IL‐1β without altering inflammation‐associated gene networks or inflammasome activation. This process occurs because macrophage exposure to LPS results in a pronounced increase in the levels of ROS, which impair IL‐1β mRNA expression [166]. In addition, serine, in conjunction with glucose and methionine, fuels the generation of SAM, which in turn supports histone H3 lysine 36 trimethylation (H3K36me3) to promote LPS‐induced IL‐1β production, thereby driving inflammatory macrophages [73]. Finally, exogenous serine restriction can also enhance antiviral innate immunity. Serine deficiency in macrophages inhibits SAM‐mediated H3K27me3 to increase ATP6V0d2 expression. ATP6V0d2 further promotes YAP lysosomal degradation to attenuate the YAP‐mediated inhibition of the TBK1–IRF3 axis, thus enhancing the IFN‐β‐mediated antiviral innate immunity of macrophages [167]. Collectively, these studies elucidate the complexity and importance of serine metabolism in regulating macrophage function and immune responses.

#### 6.3.2 Arginine and Methionine

Arginine promotes the sustained efferocytosis of anti‐inflammatory macrophages [168]. Efferocytosis is a physiological process in which apoptotic cells are cleared by phagocytes. This process helps prevent the spread of inflammation and necrosis and is essential for maintaining tissue homeostasis. Anti‐inflammatory macrophages highly express ARG1, and when apoptotic cell‐derived arginine is taken up by anti‐inflammatory macrophages, ARG1 converts it to ornithine. Ornithine can subsequently be further metabolized to putrescine by ODC, which increases the expression of Dbl (gene name Mcf2) by stabilizing the mRNA of Mcf2, a guanosine exchange factor that promotes the exchange of GDP for GTP, thereby activating the actin regulator Rac1 to facilitate subsequent cytosolic burial [67].

Additionally, during the processing of apoptotic cells, macrophages are able to convert released methionine into SAM, which provides a substrate for DNA methyltransferase 3A‐mediated DNA methylation. This epigenetic modification is capable of influencing gene expression, including the inhibition of dual‐specificity phosphatase 4, thereby prolonging the phosphorylation of extracellular signal‐regulated kinases. This sustained phosphorylation state induces the expression of Ptgs2 and subsequent activation of the PGE2–TGFβ catabolic pathway, which promotes the timely resolution of inflammation and the repair of tissues following injury [79].

A separate line of investigation examined the effects of methionine restriction on macrophage inflammatory responses. Kip and colleagues subjected mouse BMDMs to culture in medium depleted of both methionine and cystine prior to LPS stimulation. Under these conditions, they observed reduced production of IL‐6 and TNF‐α, along with decreased expression of proinflammatory markers including Ccr2 and Cd38 [148]. The concurrent removal of both amino acids raises the question of whether the observed anti‐inflammatory effects stem specifically from methionine deficiency or are confounded by cysteine deprivation.

Cysteine is a rate‐limiting precursor for glutathione synthesis, a major intracellular antioxidant. Cysteine depletion can lower glutathione levels, increase oxidative stress, and thereby influence inflammatory signaling independent of methionine. The experimental design used by Kip and colleagues does not allow definitive attribution of the observed effects to methionine deficiency alone, as the simultaneous absence of cysteine could contribute to or even drive the phenotypic changes. To isolate the specific role of methionine, additional controls would be needed—for example, comparing methionine‐deficient medium supplemented with cysteine against complete medium or using specific inhibitors of methionine metabolism such as cycloleucine. Without such controls, the findings should be interpreted as demonstrating an effect of combined sulfur amino acid restriction rather than methionine deficiency per se.

These considerations highlight a broader challenge in amino acid restriction studies: nutrients are metabolically interconnected, and depleting one amino acid can have indirect effects on others. Future studies should employ more refined approaches, including isotope tracing, metabolic profiling, and targeted supplementation, to dissect the specific contributions of individual amino acids to macrophage function.

#### 6.3.3 Gamma‐Aminobutyric Acid

Gamma‐aminobutyric acid (GABA) is a naturally occurring nonprotein amino acid that plays an important regulatory role in organisms. GABA enhances succinate‐flavin adenine dinucleotide‐lysine specific demethylase 1 signaling, which in turn regulates the histone demethylation of Bcl2l11 and Dusp2. This process reduces the formation of the NLRP3–ASC–Caspase‐1 complex, which in turn decreases the level of IL‐1β produced by proinflammatory macrophages and attenuates LPS‐induced sepsis in mice [149]. However, inhibition of GABA transport proteins impedes the activation of inflammatory vesicles and increases the capacity for mitochondrial OXPHOS. Consequently, this process leads to the suppression of proinflammatory polarization in macrophages [150]. Moreover, a recent study revealed that GABA produced by B cells promotes monocyte differentiation into anti‐inflammatory macrophages and the secretion of IL‐10, which inhibits the killing function of CD8+ T cells, resulting in antitumor immunity [169]. These findings suggest that the GABA system has a complex role in regulating macrophage function; therefore, how to precisely target the GABA system for the treatment of macrophage‐associated diseases still needs to be explored in depth.

#### 6.3.4 Tryptophan

Tryptophan is an essential amino acid that is converted into a variety of biologically active substances, including indole, in the body through a series of metabolic pathways. Indole, an organic compound, is produced primarily by the intestinal flora through tryptophan metabolism. Transplantation of tryptophan‐rich microbiota into antibiotic‐treated mice resulted in increased indole production. This process effectively reduced proinflammatory macrophage polarization during colonic inflammation by binding to 5‐hydroxytryptamine receptor 2B [151]

A study of pancreatic ductal adenocarcinoma revealed the same phenomenon. In this cancer model, TAMs exhibited high aryl hydrocarbon receptor (AhR) activity. When indole binds to AhR, AhR is translocated to the nucleus, driving macrophages to acquire an immunosuppressive phenotype and promoting the expression of TGF‐α, TGF‐β, and Arg1. Importantly, when tryptophan is removed by dietary modification, the percentage of anti‐inflammatory macrophages is reduced, and tumor size is decreased, suggesting that dietary tryptophan, which is metabolized to indole via the microbiome, is a pivotal driver of the TAM immunophenotype in pancreatic ductal adenocarcinoma [74].

#### 6.3.5 Leucine

Leucine is an essential amino acid belonging to the branched‐chain amino acid family and plays a variety of important roles in human health and physiological functions. A team reported that oral administration of leucine to mice significantly reduced the mortality rate of LPS‐induced cytokine storm syndrome. Furthermore, when proinflammatory macrophages were cultured in vitro and treated with leucine, leucine promoted macrophage polarization toward the anti‐inflammatory phenotype through the activation of mTORC1/LXRα/Arg1 signaling. Anti‐inflammatory‐type macrophages, which are normally associated with anti‐inflammatory responses, effectively reduce the levels of proinflammatory cytokines, thereby attenuating the inflammatory response [152]. Importantly, however, leucine intake requires careful regulation, as its overconsumption can result in the activation of mTORC1, leading to the phosphorylation of autophagy‐related proteins (ULK1/2 and ATG13). This process has been shown to inhibit autophagic lysosomal degradation in macrophages. This inhibition of autophagy has been shown to result in macrophage dysfunction and the promotion of foam cell formation, a hallmark of inflammatory diseases such as atherosclerosis [153]. Therefore, leucine intake must be maintained within an appropriate range, thereby ensuring a balance between its anti‐inflammatory benefits and potential risks.

#### 6.3.6 Citrulline

Citrulline is a nonessential amino acid that is naturally synthesized in the liver and intestines. A recent study revealed that citrulline is an age‐related metabolite. Citrulline supplementation effectively mitigated age‐related phenotypes, including cellular senescence, DNA damage, cell cycle arrest, and inflammatory cytokine levels. Mechanistically, citrulline acts as a potential inhibitor of mTOR activation in macrophages and reduces the mTOR–HIF1α–glycolysis pathway to combat inflammation and aging [154]. These findings reveal a key regulatory role of citrulline in macrophage metabolism to counter aging and inflammation. Another study indicated that macrophages exposed to LPS and/or IFN‐γ stimulation have reduced citrulline levels. This effect is mediated by a STAT1‐mediated increase in ASS1 expression and JAK2‐induced ASS1 phosphorylation, resulting in the conversion of citrulline into argininosuccinate. High levels of citrulline prevent proinflammatory macrophage activation and antibacterial host defense by suppressing JAK2–STAT1 signaling [96]. Taken together, these findings suggest that citrulline serves as an important regulator of macrophage activation and function.

#### 6.3.7 α‐Ketoglutarate

α‐KG, a key molecule in the Krebs cycle, plays an important role in macrophage polarization. α‐KG, which is produced by glutaminolysis, has a dual regulatory effect on macrophage activation. On the one hand, α‐KG supports anti‐inflammatory activation by promoting the Jmjd3‐dependent demethylation of H3K27 at the promoters of anti‐inflammatory marker genes, including Arg1, Ym1, Retnla, and Mrc1. In contrast, α‐KG prevents proinflammatory activation by suppressing IKKβ activation, which is mediated by the PHD‐dependent proline hydroxylation of IKKβ [69]. In addition, one study reported that in a model of liver ischemia/reperfusion injury, increased serum levels of α‐KG, which were derived from gut microbiota‐derived glutamine, promoted anti‐inflammatory macrophage polarization via the OXPHOS pathway [170]. α‐KG present in exosomes secreted by adipocytes has also been shown to increase the number of anti‐inflammatory macrophages and enhance DNA demethylation in macrophages to alleviate adipose tissue inflammation in obese mice [155]. In cholangiocarcinoma, the succinylation of PDHA1, a key enzyme in the TCA cycle, enhances PDHA1 activity and drives metabolic reprogramming, leading to the accumulation of α‐KG in the TME. This accumulation activates the OXGR1 receptor on macrophages, triggering the MAPK signaling pathway and inhibiting the expression of MHC‐II molecules, which in turn impairs macrophage antigen presentation and promotes immune suppression [156]. Thus, due to the different regulatory functions of α‐KG on macrophages, it plays various roles in inflammation‐related diseases.

Collectively, a wealth of evidence has demonstrated that both cell‐intrinsic metabolic circuits and cell‐extrinsic microenvironmental signals coordinately dictate the metabolic status, polarization, and functional output of macrophages. Dysregulation of these metabolic processes often disrupts macrophage homeostasis, thereby contributing to the initiation and progression of multiple human diseases. In the next section, we summarize the critical roles of macrophage metabolic reprogramming in representative metabolic disorders, inflammatory diseases, and other pathological conditions.

## 7 Macrophage Metabolism in Human Diseases

**FIGURE 4:**
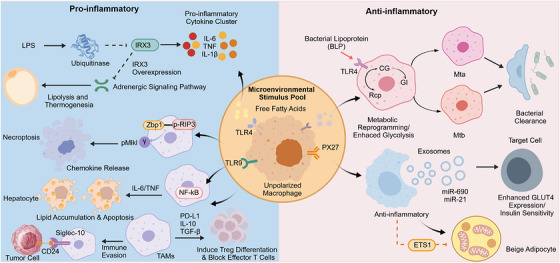
Regulation of host metabolic homeostasis and disease by macrophages. This integrated schematic diagram comprehensively depicts the metabolic reprogramming and functional remodeling undergone by unpolarized macrophages upon sensing disease microenvironmental stimuli, alongside their decisive influence on disease outcomes. As illustrated on the left, in the proinflammatory/pathogenic pathway, within the obese microenvironment, LPS and FFAs activate IRX3, promoting the release of proinflammatory factors while inhibiting adrenergic signaling in adipocytes, thereby blocking lipolysis and thermogenesis. Zbp1 phosphorylates RIP3 and activates the pMlkl axis, mediating necrotic apoptosis and chemokine release. The grancalcin/TLR9/NF‐κB axis drives the release of proinflammatory factors, leading to hepatic lipid accumulation and apoptosis, thereby accelerating the progression of MASLD. Within the TME, the Siglec‐10/CD24 axis mediates immune evasion by TAMs. High expression of PD‐L1, IL‐10, and TGF‐β induces Treg differentiation while impairing effector T‐cell function. Anti‐inflammatory/protective pathways on the right: BLP or cGAS–STING pathways initiate metabolic reprogramming, enhancing glycolysis and inducing antibacterial Mta/Mtb macrophage subpopulations to clear pathogens. Anti‐inflammatory macrophages secrete exosomes containing miR‐690 and miR‐21 to target cells, increasing GLUT4 expression and insulin sensitivity. By inhibiting ETS1 or activating TFEB, they promote mitochondrial biogenesis and beige fat thermogenesis, thereby maintaining homeostasis.

Macrophages sit at the intersection of immunity and metabolism, defending against pathogens while maintaining tissue integrity and metabolic homeostasis. Their dysregulation is now linked to a wide range of diseases—obesity, T2D, MASLD, infections, autoimmune disorders, and cancer. In the sections below, we examine how the metabolic and signaling principles outlined earlier manifest across these conditions (Figure 4). Each disease has unique features, but common themes recur: metabolic reprogramming, cell‐extrinsic signals, and tissue‐specific factors converge to shape macrophage phenotypes and disease outcomes. Identifying these shared mechanisms may reveal therapeutic strategies with broader applications.

### 7.1 Obesity

Macrophages play a central role in obesity and metabolic homeostasis, displaying remarkable functional plasticity. On one hand, they drive metabolic inflammation and contribute to obesity development through specific gene regulation. The macrophage‐specific transcription factor IRX3 illustrates this point: it promotes proinflammatory gene expression while simultaneously suppressing adrenergic signaling in adipocytes and blocking lipolysis and thermogenesis. LPS enhances IRX3 stability by inhibiting its ubiquitination, further amplifying inflammatory responses. Conversely, bone marrow‐specific IRX3 knockout protects mice from diet‐induced obesity by enhancing adaptive thermogenesis [171, 172].

IRX3 exemplifies a broader principle emerging from recent studies: tissue macrophages employ lineage‐specific transcription factors to integrate inflammatory and metabolic programs. Similar to how PPARγ coordinates lipid metabolism in anti‐inflammatory macrophages and HIF‐1α drives glycolytic reprogramming in proinflammatory cells, IRX3 serves as a molecular node linking macrophage activation states to systemic metabolic outcomes. This concept of macrophage‐intrinsic transcriptional control of whole‐body metabolism extends beyond IRX3—other factors such as CREBZF in adipose tissue macrophages and FoxO1 in KCs similarly modulate insulin sensitivity and hepatic lipid handling through mechanisms discussed in subsequent sections [173, 174].

On the other hand, anti‐inflammatory macrophages help maintain metabolic homeostasis. Adipocytes and anti‐inflammatory macrophages engage in metabolic crosstalk through mitochondrial transfer, a process impaired in obese individuals [175]. Mechanistically, anti‐inflammatory macrophages can directly promote beige adipocyte differentiation independent of the sympathetic nervous system. Under cold exposure or other stimuli, these macrophages suppress the transcription factor ETS1 in adipocytes, promoting mitochondrial biogenesis and inhibiting mitochondrial degradation. This increases thermogenic gene expression, oxygen consumption, and thermogenic capacity [176]. Activating this pathway to promote beige fat formation may offer a strategy for increasing energy expenditure and improving obesity‐related metabolic complications.

### 7.2 Type 2 Diabetes

Data from the American Diabetes Association indicate that individuals with a BMI of 30 or higher face a 2.23‐fold greater risk of developing diabetes than those with a normal BMI [177]. Saturated fatty acids play a key role in this connection. They trigger chemokine production by pancreatic islet β‐cells, which attracts circulating monocytes into the islets. These infiltrating macrophages then polarize toward a proinflammatory phenotype and secrete inflammatory factors, driving islet inflammation and β‐cell dysfunction [173].

Proinflammatory macrophages also contribute to diabetic complications. In diabetic nephropathy, lipotoxic tubular epithelial cells release extracellular vesicles that activate an inflammatory phenotype in macrophages through TGFβ signaling. These activated macrophages in turn release their own extracellular vesicles, promoting apoptosis of injured tubular epithelial cells via a death receptor 5‐dependent pathway and worsening renal injury [178].

Exosomes have emerged as important mediators of metabolic crosstalk. When mouse macrophages polarize toward an anti‐inflammatory phenotype, they secrete exosomes enriched in microRNA‐690. Injecting these exosomes into high‐fat diet‐fed mice improves glucose and insulin tolerance by targeting NADK in pancreatic islet cells [179]. Similarly, human THP‐1 macrophages stimulated with IL‐4 release exosomes carrying miR‐21, miR‐99a, miR‐146b, and miR‐378a. These exosomes act on adipocytes to increase PPARγ activity and GLUT4 expression, enhancing insulin‐dependent glucose uptake. They also boost UCP1 activity and OXPHOS, promote adipocyte autophagy, improve mitochondrial function, and accelerate fatty acid catabolism, all of which help reverse hepatic steatosis. Obese mice injected with these exosomes show reduced insulin levels and improved glucose tolerance [38].

Together, these findings point to macrophages as key players in T2D, both driving disease progression through proinflammatory activation and offering therapeutic potential through anti‐inflammatory polarization. The dual role of macrophages—harmful in one state, protective in another—underscores the importance of developing strategies that selectively target pathogenic populations while preserving or enhancing their beneficial functions.

### 7.3 Metabolic Dysfunction‐Associated Steatotic Liver Disease

Macrophages take on different roles as MASLD progresses from simple steatosis to steatohepatitis (MASH), fibrosis, and eventually hepatocellular carcinoma. Their functional phenotype shifts at each stage, often with opposing effects on disease outcomes. In both MASLD patients and mouse models, the calcium‐binding protein grancalcin is markedly upregulated in macrophages. It drives proinflammatory activation through the TLR9–NF‐κB pathway, leading to the secretion of IL‐6, TNFα, and IL‐1β. These cytokines promote lipid accumulation and apoptosis in hepatocytes, fueling hepatic steatosis and inflammation [180].

As fibrosis develops, specific macrophage subsets accumulate in the liver. Single‐cell RNA sequencing has identified a population expressing SPP1, GPNMB, FABP5, and CD63 with strong profibrotic properties [181]. In MASLD mice, these macrophages show similar gene expression profiles. Excess cholesterol taken up by these cells builds up in lysosomes, triggering lysosomal stress and activating TFE3/TFEB. This upregulates early growth response factor 1, promoting profibrotic gene expression and accelerating fibrosis [182].

The body mounts counter‐regulatory responses largely through anti‐inflammatory macrophages, though their regulatory networks are complex. The transcription factor FoxO1 normally inhibits anti‐inflammatory polarization by blocking STAT6 signaling. Conditional FoxO1 knockout in bone marrow cells pushes macrophages toward an anti‐inflammatory phenotype, reducing hepatic inflammation and slowing MASH progression [174]. Macrophage‐specific XBP1 deletion has a similar effect, dampening NLRP3 inflammasome activity and reducing TGF‐β1 secretion, which alleviates steatohepatitis and inhibits hepatic stellate cell activation [183]. Anti‐inflammatory macrophage‐derived exosomes carrying miR‐411‐5p further block fibrosis by targeting calmodulin‐regulated heme‐containing protein 1 [184].

However, as MASLD advances to hepatocellular carcinoma, the protective role of anti‐inflammatory macrophages cuts both ways. While beneficial during MASH, these same cells in the TME upregulate immunosuppressive molecules, including PD‐L1, IL‐10, and TGF‐β. This impairs effector T cell function and induces regulatory T cell differentiation, creating an immunosuppressive environment that accelerates tumor progression and promotes drug resistance [185, 186].

Therefore, macrophages take on strikingly different functional states across the MASLD spectrum—proinflammatory and tissue‐damaging early on, profibrotic as disease progresses, anti‐inflammatory and reparative in response to injury, and finally immunosuppressive and protumorigenic in advanced disease. This functional diversity reflects their remarkable plasticity and highlights the challenge of designing stage‐specific therapies.

### 7.4 Infection

Macrophages are central to the initiation, progression, and resolution of inflammatory responses during infection. Upon encountering pathogens or host‐derived inflammatory signals, macrophages are recruited to sites of infection and typically polarize toward a proinflammatory phenotype that helps contain and eliminate the threat [187]. However, as infection progresses from acute to chronic stages, both the macrophage phenotype and the surrounding microenvironment undergo significant remodeling.

Eosinophilic meningitis caused by Angiostrongylus cantonensis (AC) infection illustrates this dynamic. AC infection induces substantial macrophage infiltration into the central nervous system, with marked upregulation of Z‐DNA‐binding protein 1 (Zbp1) in these cells. Mechanistically, Zbp1 binds directly to receptor‐interacting protein 3 (RIP3) and promotes its phosphorylation, which in turn increases the phosphorylation of mixed lineage kinase‐like protein (Mlkl). This Zbp1–pRIP3–pMlkl signaling axis drives programmed necrosis in macrophages, leading to the release of proinflammatory cytokines and chemokines including TNF‐α, IL‐1α, CXCL9, and CXCL10. These mediators recruit and activate other immune cells, amplifying neuroinflammation and accelerating disease progression [188].

When acute infection shifts to chronic infection, macrophages adapt to persistent pathogen stimulation. In some cases, they participate in forming granulomatous structures—organized aggregates of immune cells that wall off pathogens but can also serve as protective niches where bacteria survive and evade immune surveillance. For example, Salmonella Typhimurium persists long‐term within splenic granulomas enriched for CD11b^+^ CD11c^+^ Ly6C^+^ macrophages, establishing a reservoir for chronic infection [189].

Pathogen exposure can also induce lasting changes in macrophage function through trained immunity. In one study, BMDMs exposed to *Staphylococcus aureus* bacterial lipoprotein (BLP) gave rise to two distinct subpopulations, designated Mta and Mtb. These cells showed enhanced expression of antimicrobial genes, improved anti‐inflammatory capacity, and greater resistance to oxidative stress. Functional changes were accompanied by metabolic reprogramming, including enhanced glycolysis, altered OXPHOS, and accumulation of anti‐inflammatory metabolites. In a mouse model of sepsis, BLP‐trained macrophages reduced systemic inflammation, promoted bacterial clearance, limited organ damage, and improved survival [190].

Beyond pathogen‐derived components, probiotics and inactivated pathogenic strains can also enhance macrophage function by activating the cGAS–STING signaling pathway, boosting host resistance to infection [191]. These observations suggest that macrophage‐mediated trained immunity, coupled with metabolic reprogramming, represents a key mechanism in host defense and offers new avenues for developing therapies against infectious diseases.

### 7.5 Autoimmune Disease

Macrophages bridge innate and adaptive immunity, and their metabolic reprogramming, phenotypic polarization, and abnormal activation drive the onset and progression of autoimmune diseases. Within different autoimmune conditions, the mechanisms regulating macrophages show marked disease‐specific heterogeneity.

In systemic lupus erythematosus (SLE), exosome‐derived microRNAs play a key role in metabolic and immune regulation. miR‐122‐5p is significantly upregulated in exosomes from SLE patients and correlates with disease activity. This miRNA promotes proinflammatory macrophage polarization by targeting and inhibiting the FOXO3/NF‐κB signaling pathway, exacerbating disease progression [192].

In autoimmune uveoretinitis, macrophages drive ocular inflammation. ATP released from stressed or damaged cells acts as a danger signal, activating the purinergic receptor P2×7 on macrophages. This triggers the expression of genes involved in inflammasome activation, phagocytosis, and the complement system, initiating sterile inflammation. Activated macrophages further promote pathogenic Th17 cell infiltration, creating a vicious cycle between innate and adaptive immunity that amplifies tissue injury [193].

Chronic autoimmune inflammation can also shape macrophage development at its source. The inflammatory environment drives expansion of myeloid cells and transcriptional reprogramming of hematopoietic stem and progenitor cells. Macrophages derived from these reprogrammed precursors display a “trained immunity” phenotype—enhanced phagocytic and bactericidal capacity coupled with potent proinflammatory function. Notably, unlike conventional trained immunity models where enhanced function depends on glycolysis, these macrophages showed no glycolytic activation, and chromatin accessibility in metabolism‐related genes was markedly reduced [194]. This decoupling of the functional phenotype from metabolic activation suggests that once trained immunity is established, macrophages can maintain high proinflammatory activity without further enhancing glycolysis. Such metabolic‐phenotypic regulation offers a new perspective on macrophage activation in autoimmune disease [195].

Beyond phenotypic and metabolic changes, abnormal macrophage proliferation and interactions with the microenvironment also contribute to pathogenesis. In inflammatory bowel disease and multiple sclerosis, excessive proliferation of TRMs drives disease. The E3 ubiquitin ligase SMURF2 normally acts as a brake, inhibiting macrophage proliferation and inflammation by targeting TBK1 for phosphorylation and lysosomal degradation. However, in autoimmune inflammation, SMURF2 expression drops in TRMs, leading to uncontrolled TBK1 activation, abnormal proliferation, and tissue damage [196].

In Type 1 diabetes, macrophages secrete IL‐15, which promotes inflammatory responses in CD226^+^ B cells. The proportion of this B cell subset correlates with disease severity [197]. Together, these findings underscore that macrophages contribute to autoimmune disease through multiple interconnected mechanisms—metabolic reprogramming, cytokine secretion, and altered proliferation—all of which offer potential targets for therapeutic intervention.

### 7.6 Cancer

TAMs are the most abundant immune cells in the TME and play a central role in malignant progression. They promote tumor growth through multiple mechanisms: regulating immune evasion, driving invasion and metastasis, and remodeling the surrounding tissue.

At the level of immune evasion, tumor cells escape macrophage‐mediated phagocytosis by overexpressing surface molecules such as CD47, PD‐L1, and B2M, which bind to inhibitory receptors on TAMs. The interaction between Siglec‐10 on TAMs and CD24 on tumor cells has emerged as a key innate immune checkpoint, creating a barrier to antitumor immunity [198]. TAMs themselves also show signs of functional exhaustion. PD‐1 expression on TAMs increases with tumor stage and correlates negatively with phagocytic capacity. Lineage tracing suggests that these PD‐1^+^ TAMs originate mainly from MDMs that infiltrate the tumor core as the disease progresses [199]. In breast cancer, CXCR4^+^ macrophages reside in the stem cell niche and enhance the survival of tumor‐initiating cells while promoting early immune escape through regulatory T cells. Their gene expression signature correlates with poor patient prognosis [200].

TAMs also drive tumor invasion and metastasis by shaping an immunosuppressive microenvironment. For example, TAMs highly express FcεR receptors, and their interaction with regulatory T cells reinforces immunosuppression, weakening antitumor surveillance and enabling metastasis [201]. Beyond immune modulation, TAMs remodel the extracellular matrix and establish premetastatic niches. In high‐grade serous ovarian cancer, TAM‐derived factors including FLT3L, leptin, and HB‐EGF activate the JAK2/STAT3 pathway, upregulating matrix metalloproteinase‐9. This promotes tumor spheroid disaggregation and cancer cell dissemination through collagen‐rich matrices, accelerating local invasion and peritoneal metastasis [202].

In colorectal cancer liver metastasis, tumor cells use RNF32 to ubiquitinate GSK3β and activate Wnt/β‐catenin signaling, which upregulates CCL2 and recruits SPP1^+^ macrophages through the CCL2/CCR2 axis. Once recruited, these macrophages bind CD44 on tumor cells, creating a positive feedback loop that sustains Wnt activation, enhances tumor stemness, and promotes metastatic colonization [203].

In short, TAMs support malignant progression through multiple interconnected pathways—immune checkpoint regulation, microenvironment remodeling, and promotion of invasion and metastasis. Targeting these cells and their signaling networks holds promise for cancer immunotherapy.

Across the diverse diseases discussed above, several common patterns emerge. Metabolic flexibility allows macrophages to adopt pathogenic phenotypes in chronic disease just as it enables rapid responses to acute infection. Context determines outcome: fructose promotes inflammation systemically but suppresses it in tumors; succinate drives proinflammatory effects in some tissues while supporting anti‐inflammatory programs in others. Cell‐intrinsic and cell‐extrinsic factors are inextricably linked: the tissue microenvironment shapes macrophage metabolism, and in turn, macrophage‐derived metabolites and exosomes influence surrounding cells. Temporal dynamics matter: TAMs can be antitumor in early‐stage cancer but protumor in advanced disease; macrophages in MASLD shift from proinflammatory to profibrotic to immunosuppressive as pathology progresses. Recognizing these patterns—rather than cataloging isolated observations—is essential for developing stage‐specific therapeutic strategies.

## 8 Therapeutic Modulation of Macrophage Metabolism

Macrophages integrate microenvironmental signals through coordinated activation of signaling pathways and metabolic reprogramming, giving rise to distinct functional phenotypes that drive diverse pathological processes—including inflammation, fibrosis, tumorigenesis, and metabolic disorders. Targeting these pathways to remodel macrophage metabolism has therefore emerged as a promising therapeutic strategy. This section systematically reviews preclinical and clinical advances in targeting macrophage metabolism, organized by therapeutic strategy and molecular target (Table 4).

**TABLE 4:** Therapeutic strategies targeting macrophage metabolism: preclinical and clinical advances.

Category	Target pathway	Drug/agent	Mechanism of action	Disease/condition	Stage	NCT number
Signaling pathways	NF‐κB	TAK‐242	TLR4 inhibitor	Liver failure	II	NCT04620148
		ILB‐202	Inhibition of IκBα degradation	Inflammation	I	NCT05843799
	PI3K/AKT/mTOR	Gedatolisib	PI3K/AKT/mTOR inhibitor	Breast cancer	III	NCT05501886
		Eganelisib (IPI549)	PI3K‐γ inhibitor	Cancer	I/II	NCT03961698 NCT02637531
		Duvelisib	PI3K‐δ, γ inhibitor	Hematologic cancers	I	NCT02783625
	JAK–STAT	Tofacitinib	JAK1/3‐STAT1 inhibitor	Cutaneous sarcoidosis, rheumatoid arthritis	I/II/III	NCT03910543 NCT00976599 NCT00413699
		Ruxolitinib	JAK1/2‐STAT3 inhibitor	Epithelial ovarian cancer	I/II	NCT02713386
		Itacitinib	JAK1 inhibitor	Refractory sarcomas	I	NCT03670069
		AZD9150 (ISIS 481464)	STAT3 inhibitor	Diffuse large B‐cell lymphoma	I/II	NCT01563302
	MARK	RV568	p38α/γ inhibitor	COPD	II	NCT01475292
		Binimetinib	MAPK/MEK inhibitor	mTNBC	I/II	NCT03106415
		CC99677	MK2 inhibitor	Ankylosing spondylitis	I	NCT03554993
Metabolic pathways	NLRP3	NT0796	NLRP3 inhibitor	Neurodegenerative diseases, chronic inflammation	Preclinical	/
		DFV890	NLRP3 antagonist	Inflammatory conditions	II	NCT04382053
	Lipid	Darapladib	Lp‐PLA2 inhibitor	Fibrotic lung diseases	Preclinical	/
	Amino acid	Citrulline	mTOR–HIF‐1α–glycolysis inhibitor	Inflammatory conditions	Preclinical	/
Cell‐based therapies	Macrophages	CAR‐macrophages	Promotion of macrophage phagocytosis	Fibrosis	Preclinical	/
Cytokines and costimulatory molecules	IL‐1β	Canakinumab	IL‐1β monoclonal antibody	Hereditary periodic fever syndromes	III	NCT01327846 NCT02059291
		Rilonacept	IL‐1α and IL‐1β blocker	Recurrent pericarditis	III	NCT03737110
	CD40	Sotigalimab	CD40 agonistic antibody	Esophageal/gastroesophageal junction cancer	II	NCT03165994
		Selicrelumab	CD40 agonistic antibody	Pancreatic cancer	I	NCT02588443
		SL172154	CD40 agonistic antibody	Ovarian cancer	I	NCT04406623

*Data sources*: US National Library of Medicine ClinicalTrials.gov Database

### 8.1 Targeting Signaling Pathways

#### 8.1.1 NF‐κB Pathway

The NF‐κB signaling pathway is a master regulator of proinflammatory macrophage polarization, making it an attractive therapeutic target [204]. However, traditional IKK inhibitors have faced challenges due to systemic toxicity and off‐target effects [205]. Novel approaches aim to achieve specificity.

TAK‐242 (resatorvid) is a small‐molecule TLR4 inhibitor that blocks upstream activation of NF‐κB. In rodent models of acute liver failure, TAK‐242 markedly alleviated organ damage and systemic inflammation [206]. A Phase II clinical trial (NCT04620148) is currently underway in patients with decompensated cirrhosis associated with alcoholic hepatitis complicated by acute‐on‐chronic liver failure. This trial aims to evaluate the efficacy, safety, and pharmacokinetic characteristics of TAK‐242, with results pending publication.

A Phase II clinical trial (NCT04620148) is currently evaluating TAK‐242 in patients with decompensated cirrhosis associated with alcoholic hepatitis complicated by acute‐on‐chronic liver failure; the results are pending. ILB‐202 is an engineered extracellular vesicle formulation carrying super‐repressor IκBα, which specifically antagonizes overactivated NF‐κB signaling in inflammatory tissues while sparing basal physiological functions. A Phase I single‐ascending dose study in 18 healthy volunteers (NCT05843799) demonstrated favorable safety and tolerability, with no serious adverse events or dose‐limiting toxicity [207].

#### 8.1.2 PI3K/AKT/mTOR Pathway

The PI3K/AKT/mTOR pathway regulates macrophage polarization in a context‐dependent manner, with particular importance in TAMs).

Gedatolisib, a pan‐PI3K/mTOR inhibitor, simultaneously blocks multiple nodes in this pathway. A Phase Ib trial (NCT02684032) in HR‐positive, HER2‐negative advanced breast cancer demonstrated objective response rates of 85.2% in first‐line treatment, significantly surpassing historical controls [208]. Mechanistically, gedatolisib reverses the immunosuppressive phenotype of TAMs by inhibiting the PI3K/AKT/mTOR axis and remodeling their metabolic programs. A Phase III trial (NCT05501886) is ongoing.

Eganelisib (IPI‐549) is a highly selective PI3K‐γ inhibitor that targets a key isoform mediating myeloid cell recruitment and TAM immunosuppression. In the Phase I/Ib MARIO‐1 trial (NCT02637531), eganelisib combined with immune checkpoint inhibitors improved patient survival in advanced solid tumors. Translational analyses revealed reduced TAM infiltration and transcriptional reprogramming toward a proinflammatory, antitumor phenotype, with enrichment of IFNγ signaling and MHC Class I/II antigen presentation pathways [209]. The Phase II MARIO‐3 trial (NCT03961698) is evaluating eganelisib in combination with atezolizumab and nab‐paclitaxel in first‐line metastatic triple‐negative breast cancer (mTNBC). Paired biopsy analyses from MARIO‐3 confirmed TAM reprogramming, immune activation, and extracellular matrix reorganization, including in PD‐L1‐negative tumors. Duvelisib, a dual PI3K‐δ/γ inhibitor approved for hematologic malignancies, is being explored for its effects on the tumor immune microenvironment (NCT02783625).

#### 8.1.3 JAK–STAT Pathway

The JAK–STAT pathway exhibits bidirectional roles in macrophage polarization: JAK–STAT1/2/5 drive proinflammatory responses, while JAK–STAT3/6 mediate immunosuppressive phenotypes. This duality positions it as a valuable therapeutic target [210].

Tofacitinib, a JAK1/3 inhibitor, targets the proinflammatory JAK–STAT1 axis. In a clinical study of cutaneous sarcoidosis (NCT03910543), all 10 patients with moderate‐to‐severe skin involvement improved, with six achieving complete remission. Treatment reduced plasma levels of cytokines, chemokines, and macrophage activation markers [211, 212] Tofacitinib has also demonstrated efficacy in rheumatoid arthritis (NCT00976599, NCT00413699) [213].

Ruxolitinib, a JAK1/2 inhibitor, targets STAT3‐mediated immunosuppression in cancer. In a Phase I/II trial in high‐grade epithelial ovarian cancer (NCT02713386), ruxolitinib combined with paclitaxel and carboplatin demonstrated good tolerability and significantly improved progression‐free survival compared with historical controls.

Itacitinib, a selective JAK1 inhibitor, is being evaluated in treatment‐refractory sarcomas (NCT03670069). In the leiomyosarcoma cohort (*n* = 8), the 12‐month overall survival was 100%, with 87.5% achieving stable disease as the best response. Paired biopsy analyses revealed significant downregulation of STAT1, CCR5, NFKB1, and CXCL10, with trends toward decreased immune cell infiltration.

AZD9150 (ISIS 481464), an antisense oligonucleotide targeting STAT3, demonstrated efficacy in heavily pretreated diffuse large B‐cell lymphoma (NCT01563302), with mechanistic studies showing reduced proportions of immunosuppressive macrophages [214].

#### 8.1.4 MAPK Pathway

The MAPK pathway (p38, JNK, ERK) regulates macrophage polarization, with p38 and JNK promoting proinflammatory phenotypes and ERK supporting immunosuppressive TAM polarization.

RV568, a narrow‐spectrum p38 MAPK‐α/γ inhibitor, was evaluated in COPD patients (NCT01475292). Following 14 days of treatment, patients showed good tolerability with marked improvement in clinical symptoms and pulmonary function. Mechanistically, RV568 inhibited CXCL8 release from sputum‐derived macrophages [215].

Binimetinib, a MEK inhibitor targeting the MAPK/ERK pathway, is being studied in combination with pembrolizumab for mTNBC. Preclinical studies suggest that it reverses the immunosuppressive metabolic phenotype of TAMs by downregulating glycolysis and lactate secretion while enhancing antigen presentation [216].

CC‐99677, an irreversible covalent MK2 inhibitor (downstream of p38 MAPK), is in development for ankylosing spondylitis (NCT03554993). In macrophages, CC‐99677 reduced TTP phosphorylation and accelerated the decay of inflammatory cytokine mRNA, showing sustained TNF inhibition compared with p38 inhibitors.

### 8.2 Targeting Metabolic Pathways

Beyond signaling pathways, direct targeting of macrophage metabolic pathways is emerging as a therapeutic strategy.

#### 8.2.1 NLRP3 Inflammasome and Immunometabolites

The NLRP3 inflammasome integrates metabolic and inflammatory signals in macrophages, making it an attractive target [217].

NT‐0796, a brain‐penetrant NLRP3 inhibitor prodrug, is being evaluated for neurodegenerative diseases and chronic inflammation. The active metabolite NDT‐19795 binds the NACHT domain of NLRP3, preventing inflammasome activation. In preclinical studies, NT‐0796 reversed high‐fat diet‐induced obesity, systemic inflammation, and astrogliosis [218, 219]. DFV890, an NLRP3 antagonist, completed Phase II trials (NCT04382053) for inflammatory conditions, with results pending [220].

#### 8.2.2 Lipid Metabolism

Darapladib, an oral Lp‐PLA2 inhibitor, was evaluated in a preclinical silicosis model. Lp‐PLA2 (encoded by Pla2g7) was identified as a key driver of profibrotic MDMs through dysregulation of cardiolipin metabolism and mitophagy. In SiO_2_‐exposed mice, darapladib significantly improved lung function, reduced collagen deposition, and corrected metabolic dysfunction. While originally developed for atherosclerosis, these findings suggest potential repurposing for fibrotic lung diseases [6].

#### 8.2.3 Amino Acid Metabolism

Several amino acid metabolism pathways are being explored as therapeutic targets. Citrulline supplementation in aged mice reversed cellular senescence and inflammation by inhibiting mTOR–HIF‐1α–glycolysis signaling [154]. Serine restriction studies have revealed complex immunometabolic effects, with implications for inflammatory and antiviral immunity [72, 73, 166, 167], although clinical translation remains early.

### 8.3 Cell‐Based Therapies Targeting Macrophages

CAR‐macrophages (CAR‐Ms) represent an innovative approach to redirect macrophage phagocytic activity. A recent preclinical study engineered BMDMs with a chimeric antigen receptor targeting urokinase‐type plasminogen activator receptor (uPAR), which is highly expressed on hepatic stellate cells in the fibrotic liver. In multiple murine models of liver fibrosis (CCL_4_, DDC, and high‐fat/cholesterol/fructose diet), adoptive transfer of uPAR CAR‐Ms significantly reduced fibrosis and restored liver function. Mechanistically, CAR‐Ms modulated the hepatic immune microenvironment, recruited and presented antigens to T cells, and mounted specific antifibrotic T‐cell responses. This provides first‐in‐class evidence for CAR‐M therapy in fibrosis [221 ^,^ 222, 223].

### 8.4 Targeting Cytokines and Costimulatory Molecules

Canakinumab, an IL‐1β neutralizing antibody, blocks downstream activation of NF‐κB and MAPK/JNK pathways, inhibiting proinflammatory macrophage polarization. Phase III trials demonstrated efficacy in hereditary periodic fever syndromes (NCT02059291) and reduced cardiovascular events and lung cancer mortality in myocardial infarction patients (NCT01327846) [224, 225]. Rilonacept, an IL‐1α/β trap, is approved for recurrent pericarditis based on Phase III data (NCT03737110) [217].

CD40 agonists promote proinflammatory macrophage polarization and antitumor immunity by regulating FAO and glutamine metabolism [226]. Sotigalimab combined with neoadjuvant chemoradiotherapy for esophageal/gastroesophageal junction cancer (NCT03165994) achieved a pathologic complete response rate of 37.9% (60% in squamous cell carcinoma), with enhanced antigen‐presenting cell infiltration and T‐cell activation. Selicrelumab (NCT02588443) remodeled the TME in resectable pancreatic cancer, reducing immunosuppressive TAMs [227]. SL‐172154, a bispecific CD47 inhibitor and CD40 agonist, induced TAM reprogramming toward proinflammatory phenotypes in platinum‐resistant ovarian cancer (NCT04406623), with 22% achieving stable disease [228].

Collectively, the strategies outlined above—targeting signaling pathways, metabolic nodes, cytokines, and macrophages themselves—have shown promising preclinical and clinical activity across inflammatory diseases, fibrosis, and cancer. However, successful translation will require overcoming key hurdles: achieving cell‐type‐specific delivery to spare homeostatic macrophage functions, moving beyond the M1/M2 dichotomy to target metabolic networks rather than individual pathways and developing rational combination regimens. Addressing these challenges will be critical for realizing the therapeutic potential of macrophage metabolic targeting.

## 9 Challenges, Limitations, and Future Perspectives

Over the past decade, the field of immunometabolism has fundamentally reshaped our understanding of macrophage biology. Despite remarkable progress, several critical questions remain unresolved. First, most mechanistic insights into macrophage metabolism have been derived from reductionist in vitro systems using BMDMs or immortalized cell lines. Although these models are invaluable for dissecting molecular pathways, they fail to recapitulate the complex metabolic microenvironment in vivo—including dynamic nutrient fluctuations, physiological oxygen tensions, and intricate cellular crosstalk within tissues. Recent advances in single‐cell RNA sequencing and spatial transcriptomics have revealed an unprecedented degree of heterogeneity among TRMs; however, how this diversity arises from metabolic regulation and how it influences disease outcomes remain largely unexplored. Second, the functional significance of many immunometabolites—such as itaconate, succinate, and α‐KG—has been primarily characterized in the context of acute inflammation. Their roles in chronic disease settings, including MASLD, atherosclerosis, and cancer, are only beginning to emerge and warrant systematic investigation. Third, although the concept of trained immunity has established that innate immune cells can retain metabolic memories of prior encounters, the mechanisms by which these metabolic imprints are maintained over time and how they contribute to disease pathogenesis remain poorly understood.

Looking forward, the field is poised for transformative advances driven by technological innovation. Spatial multiomics technologies—including spatial transcriptomics and metabolomics—now enable the in situ mapping of metabolic heterogeneity within intact tissues, offering unprecedented opportunities to decipher how macrophage metabolism is shaped by the local microenvironment in both health and disease. Organoid models incorporating TRMs provide physiologically relevant platforms for studying cell–cell interactions and metabolic crosstalk under controlled conditions [229]. Precision‐cut tissue slices, which preserve native tissue architecture and cellular composition, bridge the gap between reductionist in vitro systems and complex in vivo physiology. Induced pluripotent stem cell‐derived macrophages, capable of directed differentiation into tissue‐specific subsets, offer scalable and genetically tractable models for mechanistic studies and drug screening. The integration of these cutting‐edge approaches with traditional biochemical and genetic tools promises to unravel the complexity of macrophage metabolism with unprecedented resolution.

The therapeutic implications of targeting macrophage metabolism are substantial and are increasingly being translated into clinical development. As summarized in Table 4, inhibitors of key signaling pathways—including TLR4/NF‐κB (TAK‐242, ILB‐202), PI3K‐γ (eganelisib), JAK–STAT (tofacitinib, ruxolitinib), and p38 MAPK (RV568)—have shown promise in preclinical and clinical studies for the treatment of inflammatory diseases and cancer. However, several challenges must be overcome to fully realize the potential of metabolism‐targeted immunotherapies. The context‐dependent and often opposing roles of metabolic pathways in distinct macrophage subsets necessitate precision‐targeting strategies that preserve homeostatic functions while correcting pathological activation. The development of cell type‐specific delivery systems, such as nanoparticle formulations or antibody–drug conjugates, may enable selective targeting of pathogenic macrophage populations. Moreover, the emerging recognition that macrophages exist along a continuum of activation states—rather than as discrete M1/M2 phenotypes—calls for more nuanced therapeutic approaches that modulate metabolic networks instead of merely inhibiting or activating individual pathways. Ultimately, the integration of fundamental metabolic insights with advanced therapeutic platforms holds the promise of transforming the treatment landscape for the myriad diseases in which macrophages play central pathogenic roles.

## 10 Conclusions

The metabolic regulation of macrophages has emerged as a central theme in immunology, with far‐reaching implications for understanding disease pathogenesis and developing novel therapeutics. It is now clear that metabolic reprogramming is not merely a passive consequence of macrophage activation but an active determinant of cellular fate and function. This review has synthesized current knowledge on how intracellular metabolic pathways—glycolysis, the TCA cycle, OXPHOS, fatty acid metabolism, and amino acid utilization—are dynamically rewired during macrophage polarization and how these metabolic changes are orchestrated by core signaling cascades, including NF‐κB, PI3K/AKT/mTOR, JAK–STAT, and MAPK. We have also highlighted the emerging paradigm of cell‐extrinsic metabolic regulation, demonstrating that exogenous metabolites such as succinate, itaconate, lactate, and various amino acids act as signaling molecules that shape macrophage phenotypes through receptor‐mediated mechanisms and epigenetic modifications. Furthermore, the tissue‐specific metabolic signatures of resident macrophage populations—AMs, KCs, microglia, and TAMs—underscore the profound influence of the local microenvironment in programming cellular metabolism. Together, these insights establish a conceptual framework in which metabolism serves as an integrative hub that translates diverse environmental cues into appropriate immune responses. As the field continues to evolve, the convergence of technological innovation, mechanistic insight and therapeutic development will undoubtedly unlock new opportunities to harness the metabolic plasticity of macrophages for the benefit of human health.

## Author Contributions

**Shan Huang**: conceptualization, writing – original draft, and writing – review and editing. **Shanshan Liu**: conceptualization, funding acquisition, writing – review and editing, and supervision. **Yu Zhang**: visualization. **Jiali Min**: visualization. **Jiahui Yang**: visualization. **Yuchen Li**: visualization. **Hao Zhang**: visualization. All authors have read and approved the final manuscript.

## Ethics Statement

This article does not contain any studies with human or animal subjects.

## Conflicts of Interest

The authors declare no conflicts of interest.

## Data Availability

No data were used for the research described in the article.
